# The faunal drugstore: Animal-based remedies used in traditional medicines in Latin America

**DOI:** 10.1186/1746-4269-7-9

**Published:** 2011-03-07

**Authors:** Rômulo RN Alves, Humberto N Alves

**Affiliations:** 1 Departamento de Biologia, Universidade Estadual da Paraíba, Avenida das Baraúnas, Campina Grande, Paraíba 58109-753, Brasil; 2 Prefeitura Municipal de João Pessoa, Escola Municipal Arnaldo de Barros Moreira, Rua Capitão Francisco Pereira, 365, Bairro dos Novais, João Pessoa, Paraíba, 58088-530, Brasil

## Abstract

Zootherapy is the treatment of human ailments with remedies made from animals and their products. Despite its prevalence in traditional medical practices worldwide, research on this phenomenon has often been neglected in comparison to medicinal plant research. This review discusses some related aspects of the use of animal-based remedies in Latin America, identifies those species used as folk remedies, and discusses the implications of zootherapy for public health and biological conservation. The review of literature revealed that at least 584 animal species, distributed in 13 taxonomic categories, have been used in traditional medicine in region. The number of medicinal species catalogued was quite expansive and demonstrates the importance of zootherapy as an alternative mode of therapy in Latin America. Nevertheless, this number is certainly underestimated since the number of studies on the theme are very limited. Animals provide the raw materials for remedies prescribed clinically and are also used in the form of amulets and charms in magic-religious rituals and ceremonies. Zootherapeutic resources were used to treat different diseases. The medicinal fauna is largely based on wild animals, including some endangered species. Besides being influenced by cultural aspects, the relations between humans and biodiversity in the form of zootherapeutic practices are conditioned by the social and economic relations between humans themselves. Further ethnopharmacological studies are necessary to increase our understanding of the links between traditional uses of faunistic resources and conservation biology, public health policies, sustainable management of natural resources and bio-prospecting.

## Introduction

Throughout human history, people have used various materials from nature to cure their illnesses and improve their health [1]. Traditional human populations have a broad natural pharmacopoeia consisting of wild plant and animal species. According to the World Health Organization, 80 percent of the developing world's rural population depends on traditional medicines for its primary healthcare needs [2]. In many parts of the world, traditional medicine is the preferred form of health care, and remains the most available and affordable form of therapy in low income countries [3]. Ingredients sourced from wild plants and animals are not only used in traditional medicines, but are also increasingly valued as raw materials in the preparation of modern medicines and herbal preparations. Nature has been the source of medicinal agents for thousands of years, and an impressive number of modern drugs have been isolated from natural sources, many based on their use in traditional medicine [1].

The use of biological resources for various therapies has been documented in many different parts of the world [4-16]. Plants and animals have been used as medicinal sources since ancient times [1,2,17-22], and even today animal and plant-based pharmacopeias continue to play an essential role in world health care [1,2,4]. Although plants and plant-derived materials make up the majority of ingredients used in most traditional medical systems globally, whole animals, animal parts, and animal-derived products (e.g., urine, fat, etc.) also constitute important elements of the Materia Medica [2,23-25].

The use of organs or parts of animals as medicine is the basis of many traditional therapeutic practices [25]. Zootherapy is the treatment of human ailments with remedies made from animals and their products [2]. As Marques [26] states, "all human culture which presents a structured medical system will utilize animals as medicines". The phenomenon of zootherapy is marked both by a broad geographical distribution and very deep historical origins. In modern societies, zootherapy constitutes an important alternative among many other known therapies practiced worldwide [2,27-39]. Despite its prevalence in traditional medical practices worldwide, research on this phenomenon has often been neglected in comparison to medicinal plant research [2]. Traditionally, medicinal animals have received little attention from ethnobiologists and anthropologists. It is only within the past few decades that researchers have begun to systematically investigate the uses of medicinal plants, and an awareness of the variety and importance of nonbotanical remedies (of animal and mineral origin) is emerging [40]. In spite of the recent surge in publications about zootherapeutics the subject is still far from being well covered, and even more distant from being exhausted. The lack of zootherapeutic studies in Latin America (and in the world in general) has contributed to an underestimation of the importance of zootherapeutic resources [41,42].

Many cultures still employ traditional medicine that includes animal-derived remedies. Probably the most famous of these are the Chinese, who use animals for a variety of ailments. Lesser known and studied, though just as varied and rich is Latin America's long tradition of animal-remedies for all kinds of ailments. Latin America's rich biological and cultural diversity makes it an exceptional location in which to examine and increase our knowledge of faunistic resources used as in traditional folk medicine, to draw attention to their importance in public health, and protect traditional knowledge and biodiversity.

Latin America is outstanding both because of its great wealth of genetic resources and complex cultural diversity [43-46]. The adaptation of the various human groups to the region's rich biological resources generated invaluable local knowledge systems that include extensive information on plant and animal uses in general [43,47-59]. In that context, the aim of this study was to provide an overview of the use of medicinal animals in Latin America, identify those species used as folk remedies, and discuss the implications of zootherapy for public health and biological conservation.

## Methods

### Study area

Latin America is a vast region spanning parts of North America, almost all of South America, and much of the West Indies. It encompasses 19 countries as well as Puerto Rico, a commonwealth territory of the United States, and, arguably, even parts of southwestern United States [46]. In Latin America and the Caribbean, the population stood at 577 million in 2008 and is projected to increase to 778 million by 2050 [60]. It is the most urbanized region in the developing world, with around three-quarters of the population living in urban areas [43]. The population of Latin America is a composite of ancestries, ethnic groups, and races, making the region one of the most--if not the most--racially and ethnically diverse in the world. The specific composition varies from country to country: Some countries have a predominance of a mixed population, in others people of Amerindian origin are a majority, some are dominated by inhabitants of European ancestry, while others are primarily of African descent. Most or all Latin American countries also have large Asian minorities. Europeans are the largest single group, and they and people of part-European ancestry combine to make up approximately 80% of the population of the subcontinent [61].

Latin America is one of the world's principal culture regions. It is distinguished from other world regions by a set of common cultural traits that include language, religion, social values, and civic institutions deriving principally from the Iberian Peninsula. Spanish and Portuguese are predominant languages. Catholicism is practiced by a vast majority of the region's inhabitants, and social customs and civic institutions bear many similarities to those in Spain. Nevertheless, the region is not culturally monolithic. Indigenous cultures and peoples have influenced national and subnational cultures within region, affecting language, religion, music, food habits, social customs, and civic institutions. The descendents of African slaves have also influenced the region's culture, although their effects have been most pronounced in Brazil, the Caribbean, and coastal areas of Central and northern South America. The cultural impact of other immigrants, including those from Italy, Asia, the Middle East, and even a few from North America has been minor[46].

## Procedures

In order to examine the diversity of animals used in traditional medicine in Latin America, all available references or reports of folk remedies based on animal sources were examined [4,7-11,29-31,36,38,40,42,62-193]. Information was gathered from published articles, books and book chapters, theses and dissertations, undergraduate theses, as well as from reports, and abstracts available at international online databases such as Web of Science, Scopus and Google Scholar and journals' web sites. The resulting database encompassed information on species, family names, and conditions to which remedies are prescribed. Only taxa that could be identified to species level were included in the database. Scientific names provided in publications were updated according to the ITIS Catalogue of Life: 2011 Annual Checklist [194].

## Medicinal fauna of Latin America

The use of medicinal fauna in Latin America has been the focus of some ethnozoological research over the last two decades, mainly in countries such as Brazil, Mexico and Bolívia. These studies have demonstrated the importance of zootherapy to both urban and rural populations. This is not surprising, considering the rich biological resources and cultural of the region, that generated invaluable local knowledge systems that include extensive information on animal uses in general and medicinally useful species, in particular.

A review of the literature revealed that at least 584 animal species have been used in traditional medicine in Latin America (Table 1). The high number of animals used as medicine is not surprising given the important role played by wildlife as a source of medicines in different parts of the world. Nevertheless, the number is certainly underestimated since the amount of studies on the theme are very limited. It is self-evident that there is an urgent need for more studies into zootherapeutic practices in the region.

**Table 1:** Medicinal animals and its respective uses in popular medicine in Latin America

Family/Species	Conditions to which remedies are prescribed	References
**PORIFERA**		

**Spongiidae**		

*Spongia officinalis *Linnaeus, 1759	Unspecified	[124]

**CNIDARIANS**		

**Mussidae**		

*Mussismilia harttii *(Verril, 1868)	Vaginal discharge, diarrhoea	[11,149]

**Physaliidae**		

*Physalia physalia *(Linnaeus, 1758) - Portuguese-man-of-war, jellyfish	Asthma	[7-9,11,31,146]

**MOLLUSCS**		

**Ampullariidae**		

*Pomacea lineata *(Spix, 1827) - Snail, Clam	Asthma, sprains, boils, ulcer	[7-9,11,31,97,115,146,161,169]

**Megalobulimidae**		

*Megalobulimus oblongus *(Mueller, 1774) -clam	Asthma	[11,66]

**Donacidae**		

*Iphigenia brasiliana *(Lamarck, 1818) - giant coquina	Teething	[80,146]

**Loliginidae**		

*Loligo vulgaris *Lamarck, 1798	Unspecified	[80]

**Cassidae**		

*Cassis tuberosa *(Linnaeus, 1758) - Conch	Asthma	[99,146]

**Littorinidae**		

*Littorina angulifera *(Lamarck, 1822) - Periwinkle snail	Chesty cough, shortness of breath	[7-9,146]

**Lucinidae**		

*Phacoides pectinatus *(Gmelin, 1791) - Shellfish	Sexual impotence	[11,146]

**Melongenidae**		

*Pugilina morio *(Linnaeus, 1758) - Conch	Sexual impotence	[11,99,146]

**Mytilidae**		

*Mytella charruana *(Orbigny, 1842) - Mussel, Shellfish	Ophthalmological problems	[11,99,146]

*Mytella guyanensis *Lamarck (1819) - Mussel, Shellfish	Weakness	[7-9,146]

**Ostreidae**		

*Crassostrea rhizophorae *(Guilding, 1828) Mangrove oyster	Osteoporosis, pneumonia, stomach ache, cancer, flu, weakness, pain relief in injuries caused by the dorsal fin spine of a species of catfish, anaemia, tuberculosis	[7-9,146]

**Strombidae**		

*Aliger pugilis *Linnaeus, 1758 - West Indian fighting conch	Sexual impotence	[11,99,146]

**Teredinidae**		

*Neoteredo reynei *(Bartsch, 1920) - Shipworm	Anaemia, tuberculosis	[69,146]

*Teredo pedicellata *Quatrefages, 1849	Tuberculosis	[99,146]

**Vasidae**		

*Turbinella laevigata *(Anton, 1839) - Conch	Sexual impotence	[99,146]

**Veneridae**		

*Anomalocardia brasiliana *(Gmelin, 1791) - Clam, shellfish	Asthma, flu, stomach ache	[7-9,146]

**Octopodidae**		

*Octopus vulgaris *(Cuvier, 1799) - common octopus	Unspecified	[117]

**ANNELIDA**		

**Lumbricidae**		

*Lumbricus terrestris *(Linnaeus, 1758)	Inflamatory process	[87]

**CHELICERATA**		

**Bothriuridae**		

*Bothriurus asper *Pocock, 1893 - black scorpion	Ethnoveterinary use	[127,178]

**Buthidae**		

*Rhopalurus rochai *(Borelli 1910)	Scorpion bite, ethnoveterinary use	[127,151,178]

**CRUSTACEANS**		

**Calappidae**		

*Calappa ocellata *Holthuis, 1958 - Ocellate box crab	Asthma, osteoporosis	[11,36,146]

**Gecarcinidae**		

*Cardisoma guanhumi *Latreille, 1825 - Blue land crab	Asthma, bronchitis, wounds, boils	[11,146]

**Grapsidae**		

*Goniopsis cruentata *(Latreille, 1802) - Mangrove root crab	Epilepsy, venereal disease	[7-9,11,99]

*Plagusia depressa *(Fabricius, 1775) - Tidal spray crab	Epilepsy	[11,99,146]

**Hippidae**		

*Emerita portoricensis *Schmitt, 1935 - Puerto Rican sand crab	Earache	[11,99,146]

**Ocypodidae**		

*Ocypode quadrata *(JC Fabricius, 1787) - Ghost crab	Asthma, haemorrhage in women, flu, to alleviate the symptoms of intoxication with poison of niquim (Pisces, Batrachoididae)	[7-9,119,146]

*Ucides cordatus *(Linnaeus, 1763) - Swamp Land crab	Haemorrhage in women, incontinence urinary, osteoporosis, cough, asthma, tuberculosis, womb disorders, arthrosis, bronchitis	[7-9,119,146]

*Uca maracoani *(Latreille, 1802) - Fiddler crab	Asthma, whooping cough	[7-9,119,146]

**Palaemonidae**		

*Macrobrachium carcinus *(Linnaeus, 1758) - Bigclaw river shrimp, Painted river prawn	Amnesia	[11,99,146]

*Macrobrachium acanthurus *(Wiegmann, 1836) - Cinnamon river shrimp	Irritation when milk teeth are erupting	[7-9,146]

*Macrobrachium borellii *(Nobili, 1896) - Freshwater shrimp	Irritation when milk teeth are erupting	[7-9,146]

**Penaeidae**		

*Xiphopenaeus schmitti *(Burkenroad, 1936) - Southern white shrimp	Irritation when milk teeth are erupting, skin spots	[7-9,146]

*Xiphopenaeus kroyeri *(Heller, 1862) - Atlantic seabob	Irritation when milk teeth are erupting, skin spots	[7-9,146]

**Pseudosquillidae**		

*Cloridopsis dubia *(H. M. Edwards, 1837) - Mantis shrimp	Asthma	[7-9,146,164]

**Armadillidiidae**		

*Armadillidium vulgare *(Latreille, 1804) - Pillbug	Asthma	[177]

**Sesarmidae**		

*Aratus pisoni *(H. Milne Edwards, 1837) - Mangrove crab	Epilepsy, to alleviate the symptoms of intoxication with poison of *Colomesus psittacus*	[7,9,11,99,146]

**Mithracidae**		

*Mithrax hispidus *(J. F. W. Herbst, 1790) - coral clinging crab	Burns	[151]

**Portunidae**		

*Callinectes bocourti *A. Milne-Edwards, 1879 - Bocourt swimming crab	Unspecified	[80]

*Callinectes exasperatus *Gerstaecker, 1856 - rugose swimming crab	Unspecified	[80]

**INSECTS**		

**Apidae**		

*Apis mellifera *(Linnaeus, 1758) - Africanised honey bee	Cough, flu, rheumatism, tuberculosis, bronchits, hoarseness, ulcer, diabetes, verminosis, headache, giddiness, backache, wounds, burns, mumps, varicose veins, arthrosis, cellulitis, amoebiasis, sore throat, asthma, anaemia, catarrh	[7-9,63,97,119,146,170]

*Cephalotrigona capitata *(Smith, 1854) - Bee	Snake bite	[11,146]

*Frieseomelitta silvestrii *(Friese, 1902) - Stingless bee	Flu	[11,146]

*Frieseomelitta varia *(Lepeletier, 1836) - Bee	Gonorrhea	[136]

*Melipona compressipes *(Fabricius, 1804) - Stingless bee	Asthma, cough	[7-9,146]

*Melipona mandacaia *Smith, 1863 - Stingless bee	Wounds	[146,170]

*Melipona quadrifasciata *Lepeletier, 1836 - Neotropical stingless bee	Snake bite	[146,187]

*Melipona scutellaris *(Latreille, 1811) - Stingless bee	Headache, migraine, stroke, verminosis, stomach ache, tuberculosis, haemorrhage, cataracts, mycosis in the mouth, flu, cancer, asthma, bronchits, intestinal disorders, cough, sexual impotence, ophthalmological problems, weakness, thrombosis, amoebiasis, snake bite, rabies, sinusitis, fatigue	[7,9,98,109,110,164]

*Melipona subnitida *(Ducke, 1910) - Honey bee	Flu, sore throat	[7-9,11,109,110,118]

*Melipona fulva *(Lepeletier, 1836) - bee	Unspecified	[86]

*Melipona asilvai *Moure, 1971 - bee	Headache, flu	[121]

*Melipona marginata *Lepeletier, 1836 - bee	Cough	[81]

*Partamona Cupira *(Smith, 1863) - Stingless bee	Sore throat, swelling, headache, thrombosis, stroke, leucoma, "slightly clean", cuts, wounds, cough, catarrh, toaday, sinusitis, effusion	[11,99,109,110,128,164,165,187]

*Partamona seridoensis *Pedro & Camargo, 2003 - Cupira bee	Ethnoveterinary uses	[127,178]

*Plebeia *cf. *emerina *Friese, 1900 - Mosquito	Mycosis in the mouth area	[11,118,166,170,177]

*Tetragonisca angustula *Latreille, 1811 - Bee	Cataracts, sinusitis, cough, flu, ophthalmological problems, sore throat, leucoma	[7-9,71,112,170]

*Trigona mosquito *Lutz, 1931 - Stingless bee	Cough	[11,71,113]

*Trigona spinipes *(Fabricius, 1793) - Stingless bee	Asthma, cough, flu, bronchits, acne, diabetes, stroke, thrombosis, migraine, itching, sore throat, giddiness, weakness, scabies, nasal congestion, to induce abortion, whooping cough, irritation when milk teeth are erupting, earache, epilepsy, shortness of breath, late menstruation, fatigue, effusion	[11,97,110,115,118,161,164,170]

*Lestrimelitta limao *(Smith, 1863) - bee	Dizziness	[81]

**Forficulidae**		

*Forficula auricularia *Linnaeus, 1758	Earache, whooping cough	[38]

**Tenebrioninae**		

*Eleodes spinipes *(Solier, 1848)	To keep away from bad spirits	[103]

**Cerambycidae**		

*Macrodontia cervicornis *(Linné, 1758)	Unspecified	[82]

**Blattidae**		

*Periplaneta americana *(Linnaeus, 1758) - American cockroach	Heartburn, asthma, stomach ache, intestinal colic, earache, alcoholism, epilepsy, vomit, boil, haemorrhage, bronchits, diarrhoea, gonorrhea, panaris, cancer, stroke, burns, menstrual cramps, wounds, to suck a splinter out of skin or flesh, detoxification, avoiding pregnancy	[7,9,11,31,71,109,112,115,128,130,170]

*Eurycotis manni *(Rehn, 1916)	Unspecified	[170]

**Blaberidae**		

*Rhyparobia maderae *(Fabricius, 1781) - Madeira cockroach	Asthma	[50]

**Chrysomelidae**		

*Coraliomela brunnea *Thumberg, 1821 - Fake cockroach	Epilepsy	[8,11,132,146]

*Pachymerus *cf. *nucleorum *(Fabricius, 1792) - Caterpillar	Earache, stroke, swelling, wounds, seborrheic dermatitis, inflammation, thrombosis	[71,112,159,170]

**Curculionidae**		

*Rhynchophorus palmarum *Linnaeus, 1758 - Pest of coconut palm	Fever, headache, boils	[149,153]

*Rhinostomus barbirostris *Fabricius, 1775 - Pest of coconut palm	Fever, headache, boils	[149,153]

*Rhina barbirostris *Champion, G.C., 1910	Fever, headache, boils	[153]

**Formicidae**		

*Atta cephalotes *(Linnaeus, 1758) - Leaf-cutter ant	Sore throat	[7-9,11,66,144]

*Atta serdens *(Linnaeus, 1758) - Leaf-cutting	Stomach ache, heart diseases, chest palpations	[11,70,71,113]

*Dinoponera quadriceps *(Santschi, 1921) - Bullet ant	Asthma	[7-9,11,109,170]

*Acromyrmex landolti *(Emery, 1980)- ant	Asthma	[66]

*Solenopsis saevissima *(Smith, 1855) - Ant	Wart	[102]

**Gryllidae**		

*Acheta domesticus *(Linnaeus, 1758) - House cricket	Scabies, asthma, eczema, lithiasis, earache, oliguresis, rheumatism, urine retention, children that urinate in bed and speak with lateness, incontinence urinary, ophthalmological problems	[11,113]

*Paragryllus temulentus *Saussure 1878 - Cricket	Rheumatism	[69]

*Gryllus assimilis *(Fabricius, 1775) - cricket	Warts	[81]

**Meloidae**		

*Palembus dermestoides *(Fairmaire, 1893) - Peanut beeatle	Sexual impotence, ophthalmological problems, rheumatism, weakness	[11,71,113,163]

*Pseudomeloe andensis *(Guérin Méneville 1992)	Warts	[189]

**Muscidae**		

*Musca domestica *(Linnaeus, 1758) - House fly	Boil, baldness, eyesore, external sebaceus lamps, stye, spots in the face, ophthalmological problems, dermatosis, cysties, erysipelas	[11,115,165]

**Pediculidae**		

*Pediculus humanus *Linnaeus, 1758 - Body louse, Head louse	Tootache	[159]

**Psychidae**		

*Eurycotis manni *Rehn, 1916 - Beetle	Headache	[11,71,115,128,146,177]

*Oiketicus kirbyi *Guilding, 1827 - Case moth	Asthma, earache, haemorrhage	[11,113]

**Termitidae**		

*Microcerotermes exignus *(Hagen, 1858), Termite	Asthma, bronchits, flu, whopping cough	[11,70]

*Nasutitermes macrocephalus *(Silvestri, 1903) - Termite	Asthma, catarrh, bronchitis, 'catarrh in the chest' coughs, influenza, sore throat, sinusitis, tonsillitis and hoarseness	[128,130,144]

*Nasutitermes corninger *(Motschulsky, 1855) - termite	Unspecified	[172]

**Vespidae**		

*Apoica pallens *(Oliv. 1791) - Paper wasp	Thrombosis, ashtma, giddiness, nasal haemorrhage, haemorrhage, stroke, disorders after parturition, ophthalmological problems, mumps, late menstruation	[11,71,112,118,146,166,170]

*Brachygastra lecheguana *(Latreille, 1824) - Dark paper wasp	Cough, asthma	[11,113]

*Polistes canadensis *(Linnaeus, 1758) - Wasp	Cough, whooping cough	[11,116,165]

*Polybia sericea *(Olivier, 1791) - Wasp	Thrombosis	[11,118]

*Protopolybia exigua *(Saussure, 1854) - Wasp	Evil eye, tobaccoism, ophthalmological problems	[11,118]

*Synoeca surinama *(Linnaeus, 1767) - Paper wasp	Asthma, shortness of breath	[11,71,118]

*Protonectarina sylveirae *(Saussure, 1854)-	Mumps, hemorrhage, blooding and menstrual problems	[66]

**ECHINODERMS**		

**Echinasteridae**		

*Echinaster brasiliensis *Müller & Troschel, 1842 - Starfish	Asthma	[11,36,63,130]

*Echinaster echinophorus *Lamarck, 1816 - Starfish	Asthma	[71,99,130,163]

**Echinometridae**		

*Echinometra lucunter *(Linnaeus, 1758) - Rock boring urchin	Asthma	[11,36,99,114,119,130,146,163,228]

**Luidiidae**		

*Luidia senegalensis *Lamarck, 1916 - Starfish	Asthma, cough, metrorrhagia	[7-9,11,36,99,114,119,130,146,163,228]

**Mellitidae**		

*Mellita sexiesperforata *(Leske, 1778) - Six holed keyhole urchin	Asthma, cough	[99]

*Mellita quinquiesperforata *(Leske, 1778)	Asthma	[11,99,130]

**Oreasteridae**		

*Oreaster reticulatus *(Linnaeus, 1758) - Starfish	Asthma	[7-9,30,31,66,109,128,146]

**Toxopneustidae**		

*Lytechinus variegatus *(Lamarck, 1816) - Green sea urchin	Snake bite	[68,146]

**FISHES**		

**Auchenipteridae**		

*Trachelyopterus galeatus *(Linnaeus, 1766) - Driftwood Cat	Umbilical hernia, asthma, sexual impotence	[71,97,161,163,228]

**Anostomidae**		

*Leporinus friderici *(Bloch, 1794) - Frederici's leporinus	Earache	[188]

*Leporinus piau *Fowler, 1941 Black piau	Rheumatism	[71]

*Leporinus steindachneri *Eigenmann, 1907	Problems with cholesterol	[109]

*Schizodon knerii *(Steindachner, 1875) - White piau	Leucoma, edema	[97]

**Ariidae**		

*Bagre bagre *(Linnaeus, 1766) - Coco sea catfish	pain relief in injuries caused by the dorsal fin spine of a species of catfish	[114,115,163]

*Genidens barbus *(Lacepède, 1803) - White sea catfish	pain relief in injuries caused by the dorsal fin spine of a species of catfish	[8,114,162]

*Genidens genidens *(Cuvier, 1829) - catfish	pain relief in injuries caused by the dorsal fin spine of a species of catfish	[8,126]

*Sciadeichthys luniscutis *(Valenciennes, 1837) - Catfish	pain relief in injuries caused by the dorsal fin spine of a species of catfish	[8,114,162]

*Genidens barbus *(Lacepède, 1803 - catfish	Unspecified	[111]

*Cathorops spixii *(Agassiz, 1829) - catfish	Unspecified	[80]

**Aspredinidae**		

*Aspredo aspredo *(Linnaeus, 1758) - Banjo, catfish	Asthma	[7-9]

*Aspredinichthys tibicen *(Valenciennes, 1840) - Tenbarbed banjo	Asthma	[7-9]

**Balistidae**		

*Balistes capriscus *Gronow, 1854 - Grey triggerfish	Bronchits	[85]

*Balistes vetula *(Linnaeus, 1758) - Queen triggerfish	Stroke, asthma, thrombosis, earache, Injuries caused by fish species, haemorrhage, ascites, schistosomiasis, appendicitis, menstrual cramps, gastritis	[7-9,114,162,228]

**Batrachoididae**		

*Thalassophryne nattereri *(Steindachner, 1876) - Venomous toadfish	Injuries caused by fish species	[4,7-9,114,115,162]

**Callichthyidae**		

*Callichthys callichthys *(Linnaeus, 1758) - Armoured catfish	Asthma, umbilical hérnia	[4,8,97,114,115]

**Carcharhinidae**		

*Carcharhinus limbatus *(Müller & Henle, 1839) - Blackfin shark	Osteoporosis	[7-9]

*Carcharhinus porosus *(Ranzani, 1840) - Smalltail shark	Asthma, rheumatism, wounds, inflammations, osteoporosis, anaemia	[7-9,69,114]

*Carcharhinus leucas (*Valenciennes, 1839) - shark	Unspecified	[30]

*Galeocerdo cuvier *(Péron & Lesueur, 1822) - Tiger shark	Osteoporosis	[7-9]

*Rhizoprionodon lalandii *(Müller & Henle, 1839) - Brazilian sharpnose shark	Rheumatism	[7-9]

*Rhizoprionodon porosus *(Poey, 1861) - Sharpnose shark	Rheumatism	[7-9]

*Sphyrna lewini *(Griffith & Smith, 1834) - Scalloped hammerhead	Asthma, wounds, rheumatism, inflammation	[8,9,114,162,228]

**Salmonidae**		

*Oncorhynchus mykiss *(Walbaum, 1792) - redband trout	Rheumatism, bad smell of feet	[148]

**Centropomidae**		

*Centropomus parallelus *Poey, 1860 - Smallscale fat snook	Nephritis	[149]

*Centropomus undecimalis *(Bloch, 1792) - Common snook	Edema in the legs	[7-9,114,162,228]

**Characidae**		

*Astyanax bimaculatus *(Linnaeus, 1758) - Twospot astyanax	Alcoholism, leishmaniosis, skin burns, wounds, rheumatism	[7-9,114,157,162,228]

*Paracheirodon axelrodi *(Schultz, 1956) - Cardinal tetra	Ashtma	[82]

*Chalceus macrolepidotus *Cuvier, 1818	Unspecified	[82]

*Brycon nattereri *Günther, 1864 - Pirapitinga	Flu	[168]

*Colossoma macropomum *(Cuvier, 1818) - Black-finned colossoma	Paralysis of arms and legs	[8,70]

*Hydrolycus scomberoides *(Cuvier, 1816) - Vampire characin	Earache	[8,188]

*Salminus brasiliensis *(Cuvier, 1816)- Jaw characin	Unspecified	[179]

**Clupeidae**		

*Opisthonema oglinum *(Lesueur, 1818) - Atlantic thread herring	Alcoholism	[7-9]

**Dasyatidae**		

*Dasyatis guttata *(Bloch & Schneider, 1801) - Longnose stingray	Asthma, Injuries caused by fish species, burns	[7-9]

*Dasyatis marianae *(Gomes, Rosa & Gadig, 2000) - Brazilian large-eyed stingray	Asthma, Injuries caused by fish species, burns	[7-9])

**Doradidae**		

*Franciscodoras marmoratus *(Reinhardt, 1874) - Urutu	Injuries caused by fish species	[149]

*Lithodoras dorsalis *(Valenciennes, 1840) - Bacu Pedra	Swelling	[7-9]

*Megalodoras uranoscopus *(Eigenmann & Eigenmann, 1888) - Catfish	Rheumatism	[188]

*Platydoras costatus *(Linnaeus, 1758) - Catfish	Rheumatism	[188]

*Pterodoras granulosus *(Valenciennes, 1821) - Catfish	Rheumatism	[188]

*Oxydoras niger *(Valenciennes, 1821) - Catfish	Rheumatism	[82,188]

**Echeneidae**		

*Echeneis naucrates *Linnaeus, 1758 - Live sharksucker	Asthma, bronchits	[8,114,162,164]

**Electrophoridae**		

*Electrophorus electricus *(Linnaeus, 1766) - Electric eel	Sprains, bruises, insect bites, snake bite, asthma, flu, pain in general, muscle strain, rheumatism, osteoporosis, deafness, pneumonia, itching, tuberculosis, earache, toothache	[7-9,31,114,162,164,188]

**Erythrinidae**		

*Erythrinus erythrinus *(Bloch & Schneider, 1801) - Red (hi-fin) Wolf fish	Asthma	[97]

*Hoplias malabaricus *(Bloch, 1794) - Trahira	Ophthalmological problems, rheumatism, cataracts, wounds, snake bite, conjunctivitis, stroke, thrombosis, asthma, toothache, fever, earache, diarrhoea, deafness, boils, bleedings, Alcoholism, tetanus, sore throat, itching, sprains, leucoma	[7-9,31,63,97,114,126,162,164,188]

*Hoplias lacerdae *Miranda Ribeiro, 1908 Giant trahira	Leucoma	[71]

**Gadidae**		

*Gadus morhua *Linnaeus, 1758 - Atlantic cod	Boils, backache and rheumatism	[66,228]

**Ginglymostomatidae**		

*Ginglymostoma cirratum *(Bonnaterre, 1788) - Nurse shark	Rheumatism	[7-9]

**Heptapteridae**		

*Pimelodella brasiliensis *(Steindachner, 1876) - Mandim	Injuries caused by that fish species	[168]

**Holocentridae**		

*Holocentrus adscensionis *(Osbeck, 1765) - Squirrelfish	Wounds	[68]

**Megalopidae**		

*Megalops atlanticus *(Valenciennes, 1847) - Tarpon	Stroke, headache, asthma, shortness of breath, thrombosis, chest pain, injuries caused by bang	[7-9,97,114]

**Monacanthidae**		

*Stephanolepis hispidus *(Linnaeus, 1766) - Common filefish	Unspecified	[111]

**Muraenidae**		

*Gymnothorax funebris *Ranzani, 1840 - Green moray	Bleeding	[7-9]

*Gymnothorax moringa *(Cuvier, 1829) - Spotted moray	Bleeding	[7-9]

*Gymnothorax vicinus *(Castelnau, 1855) - Purplemouth moray	Bleeding	[7-9]

**Odontaspididae**		

*Eugomphodus taurus *(Rafinesque 1810) - Cação-mangona	Unspecified	[111]

**Engraulidae**		

*Anchoviella lepidentostole *(Fowler, 1911)	Unspecified	[80]

**Pristigasteridae**		

*Pellona flavipinnis *Valenciennes, 1836 - Yellowfin river pellona	Unspecified	[80]

**Scombridae**		

*Scomberomorus cavalla *Cuvier, 1829 - Kingfish	Unspecified	[80]

**Myliobatidae**		

*Aetobatus narinari *(Euphrasen, 1790) - Spotted eagle ray	Asthma, injuries caused by fish species, burns, haemorrhage	[7-9]

**Narcinidae**		

*Narcine brasiliensis *(Olfers, 1831) - Brazilian electric Ray	Toothache	[111,114,162]

**Ogcocephalidae**		

*Ogcocephalus vespertilio *(Linnaeus, 1758) - Batfish	Asthma, bronchits	[7-9,68]

*Ogcocephalus nasutus *(Cuvier, 1829) - Batfish	Asthma	[79]

**Loricariidae**		

*Hypostomus plecostomus *(Linnaeus, 1758)	Asthma	[79]

**Carangidae**		

*Hemicaranx amblyrhynchus *(Cuvier, 1833)	To accelerate recovery after parturition	[79]

**Osteoglossidae**		

*Arapaima gigas *(Schinz, 1822) - Giant arapaima	Asthma, pneumonia	[188]

*Osteoglossum ferreirai *(Kanazawa, 1966) - Aruanã	Dermatogical problems	[87]

**Pimelodidae**		

*Phractocephalus hemioliopterus *(Bloch & Schneider, 1801) - Redtail catfish	Asthma, wounds, hernia, burns in the skin, rheumatism, flu, cough, pneumonia	[87,188]

*Pseudoplatystoma corruscans *(Spix & Agassiz, 1829) - Spotted sorubim	Flu	[168]

*Pseudoplatystoma fasciatum *(Lunnaeus, 1776) - Tiger catfish	Cold	[188]

*Pseudoplatystoma tigrinum *(Valenciennes, 1840) - Catfish	Unspecified	[179]

*Sorubimichthys planiceps *(Spix & Agassiz, 1829)	Leishmaniosis, tuberculosis	[188]

*Zungaro zungaro*((Humboldt, 1821) - Black manguruyu	Asthma, toothache, earache, wounds, athlete's foot, burns in the skin, rheumatism, flu	[188]

*Rhamdia quelen *(Quoy & Gaimard, 1824)	Tonic	[177]

**Potamotrygonidae**		

*Paratrygon aiereba *(Müller & Henle, 1841) - Discus Ray	Asthma, hernia, flu, pneumonia, cough, earache, burns	[188]

*Potamotrygon hystrix *(Müller & Henle, 1834) - Porcupine river stingray	Asthma, hernia, flu, pneumonia, cough, earache, burns	[70,188]

*Potamotrygon motoro *(Müller & Henle, 1841) - Ocellate river stingray	Asthma, hernia, flu, pneumonia, cough, earache, burns	[188]

*Potamotrygon orbignyi *(Castelnau, 1855)	Injuries caused by fish species	[7-9]

*Plesiotrygon iwamae *Rosa, Castello & Thorson, 1987	Injuries caused by fish species, wounds, cracks in the sole of the feet	[7-9]

*Potamotrygon signata *German, 1913	Unspecified	[80]

***Pristidae***		

*Pristis pectinata *Latham, 1794 - Smalltooth sawfish	Asthma, rheumatism, arthritis	[7-9]

*Pristis perotteti *Müller & Henle, 1841 - Largetooth sawfish, espadarte	Asthma, rheumatism and arthritis	[7-9]

**Prochilodontidae**		

*Prochilodus argenteus *Spix & Agassiz, 1829 -	To avoid swelling of the breast feeding, mycosis	[168]

*Prochilodus lineatus *(Valenciennes, 1836)	Unspecified	[160]

*Prochilodus nigricans *Spix & Agassiz, 1829 - Black prochilodus	Chilblain, skin burns, wounds, rheumatism, eye pains	[157,188]

*Prochilodus marggravii *(Walbaum, 1792)	Unspecified	[168]

**Rajidae**		

*Atlantoraja cyclophora *Regan, 1903 - Eyespot skate	Haemorrhage after delivery	[68]

**Serrasalmidae**		

*Mylossoma duriventre *(Cuvier, 1818)	Venereal disease	[188]

*Serrasalmus brandtii *(Lütken, 1875) - White piranha	Inflammations, sexual impotence	[71,97,126]

*Serrasalmus rhombeus *Linnaeus 1766	Unspecified	[80,82]

**Sciaenidae**		

*Cynoscion acoupa *(Lacepède, 1801) - Acoupa weakfish	Renal failure	[7-9,31]

*Cynoscion leiarchus *(Cuvier, 1830) - Smooth weakfish	Renal failure	[7-9,31]

*Micropogonias furnieri *(Desmarest, 1823) - Whitemouth croaker	Injuries caused by fish species, cough, asthma, bronchits	[8,85,114,162]

*Pachyurus francisci *(Cuvier, 1830) - San Francisco croaker	Asthma, urinary incontinence, backache	[168]

*Plagioscion surinamensis *(Bleeker, 1873) - Bashaw	Urinary disorders, haemorrhage, snake bites	[188]

*Plagioscion squamosissimus *(Heckel, 1840) - South american silver croaker	Urinary disorders, haemorrhage, snake bites	[188]

**Sparidae**		

*Calamus penna *(Valenciennes, 1830) - Sheepshead porgy	Asthma	[162]

**Synbranchidae**		

*Synbranchus marmoratus *Bloch, 1795 - Marbled swamp eel	Bronchits	[114,162]

**Syngnathidae**		

*Hippocampus erectus *Perry, 1810 - Horsefish	Asthma	[7-9,31,75]

*Hippocampus reidi *(Ginsburg, 1933) - Longsnout seahorse	Asthma, edema, bronchits, haemorrhage, haemorrhage in women, disorders after parturition, gastritis, tuberculosis, to prevent abortion	[7-9,31,63,68,85,164]

*Hippocampus ingens *Girard, 1858	Unspecified	[76]

*Hippocampus spinosissimus *Weber, 1913	Unspecified	[76]

*Hippocampus trimaculatus *Leach, 1814	Unspecified	[76]

**Tetraodontidae**		

*Colomesus psittacus *(Bloch & Schneider, 1801) - Banded puffer	Breast cancer, backache, warts	[7-9,31]

*Sphoeroides testudineus *(Linnaeus, 1758) - Checkered puffer	Rheumatism	[63,162]

**Trichiuridae**		

*Trichiurus lepturus *Linnaeus, 1758 - Largehead hairtail	Asthma	[8]

**Gymnotidae**		

*Gymnotus carapo *Linnaeus, 1758 - Banded knifefish	Unspecified	[179]

**Urolophidae**		

*Urotrygon microphthalmum *(Delsman, 1941) - Smalleyed round stingray	Asthma, Injuries caused by fish species, burns	[7-9,31]

**AMPHIBIANS**		

**Bufonidae**		

*Rhinella schneideri *(Werner, 1894) - Cururu toad	Urinary incontinence, dental caries, cancer, wounds, boils, erysipelas acne, to induce abortion	[97,162]

*Rhinella marina *(Linnaeus, 1758) - toad	Scorpion sting, erysipelas	[87,152,158]

*Rhinella jimi *(Stevaux, 2002)- toad	Gastritis, cancer	[81]

*Rhinella icterica *(Spix, 1824)	Unspecified	[80]

*Bufo bocourti *Brocchi, 1877 - toad	Rabies, AIDS	[38]

*Bufo macrocristatus *Firschein and Smith, 1957 - toad	Unspecified	[38]

*Incilius valliceps *(Wiegmann, 1833)	Unspecified	[125]

**Leptodactylidae**		

*Leptodactylus labyrinthicus *(Spix, 1824) - South american pepper frog	Earache, rheumatism, joint pain, cancer, sore throat	[97,162]

*Leptodactylus vastus *Lutz, 1930-frog	Sore throat	[66]

*Leptodactylus troglodytes *(A. Lutz, 1926) - Jia	Unspecified	[4]

*Eleutherodactylus laticeps *(Duméril, 1853)	Unspecified	[38]

*Eleutherodactylus glaucus *Lynch, 1967	Unspecified	[38]

**Ranidae**		

*Rana maculata *Brocchi, 1877	Rabies	[38]

*Rana berlandieri *Baird, 1859		[38]

*Lithobates montezumae *(Baird, 1854)	Unspecified	[125]

*Lithobates spectabilis *(Hillis and Frost, 1985)	Unspecified	[125]

**Hylidae**		

*Hyla chaneque *Duellman, 1961	Unspecified	[38]

*Hyla venulosa *(Laurenti, 1768)		[60]

*Phyllomedusa bicolor *(Boddaert, 1772)	Rheumatism, diabetes	[122]

*Trachycephalus resinifictrix *(Goeldi, 1907)	Unspecified	[82]

*Phyllomedusa burmeisteri *Boulenger, 1882	Unspecified	[183]

**Microhylidae**		

*Hypopachus barberi *Schmidt, 1939	Unspecified	[38]

**Ceratophryidae**		

*Telmatobius culeus *(Garman, 1876)	Impotence	[180]

**REPTILES**		

**Liolaemidae**		

*Liolaemus pantherinus *Pellegrin 1909	Fractures, wounds	[65,189]

*Liolaemus alticolor *Barbour 1909	Fractures, wounds	[189]

**Gekkonidae**		

*Hemidactylus mabouia *(Moreau de Jonnes, 1818) - Afro-American house gecko	Sore throat	[7-9,31,164])

*Gonatodes hasemani *Griffin 1917	Unspecified	[174]

**Iguanidae**		

*Iguana iguana *(Linnaeus, 1758) - Common iguana	Earache, erysipelas, asthma, rheumatism, edema, abscesses, joint pain, wounds, acne, athlete's foot, sore throat, swelling, burn, tumour, to suck a splinter out of skin or flesh, boil, injuries caused by the spines of the 'arraia' and others fishes, inflammation, hernia, body aches, corisa, womb disorders, menstrual cramps	[7-9,31,97,108,164,166])

*Ctenosaura pectinata *Wiegmann 183	Unspecified	[94,105]

*Ctenosaura similis *GRAY 1831	Unspecified	[77]

**Polychrotidae**		

*Polychrus acutirostris *Spix 1825	Impotence	[126]

*Polychrus marmoratus *Linnaeus 175	Impotence	[126]

*Norops fuscoauratus *D'orbigny 1837	Unspecified	[174]

**Teiidae**		

*Ameiva ameiva *(Linnaeus, 1758) - Lizard	Inflammation, dermatitis, venereal diseases, snake bites	[87,97,108,174]

*Kentropyx pelviceps *Cope 1868	Unspecified	[174]

*Cnemidophorus *gr. *ocellifer *(Spix, 1825) - Lizard	Inflammation, dermatitis, venereal diseases, snake bites	[97,108]

*Tupinambis merianae *(Duméril & Bibron, 1839) - Lizard	Earache, deafness, rheumatism, erysipelas, skin thorns and wounds, respiratory diseases, sore throat, snake bite, asthma, tumour, swelling, infection, bronchits, perforation, oftalmological problems	[7-9,31,85,97,108,111,119,162,164,166,173,190]

*Tupinambis teguixin *(Linnaeus 1758) - Lizard	Sexual impotence, rheumatism, erysipelas, dermatitis, snake bites, asthma, tetanus, earache, thrombosis, wounds, panaris, swelling, herpes zoster, irritation when milk teeth are erupting, jaundice, inflammation, tumour, sore throat, infection, bronchits, injuries caused by the spines of the 'arraia', pain relief in injuries caused by snake bites, toothache, suck a splinter out of skin or fresh, headache, cough, stroke, coarse throat	[7-9,31,68,97,108,157,161,164,166]

*Tupinambis rufescens *Günther 1871 - tegu	Cuts, snake bites, cold	[120]

**Phrynosomatidae**		

*Sceloporus serrifer *Cope 1866	Unspecified	[38]

*Sceloporus taeniocnemis *Cope 1885	Unspecified	[38]

*Sceloporus acanthinus *Bocourt 1873	Unspecified	[91]

*Sceloporus spinosus *Wiegmann 1828	Unspecified	[91]

*Sceloporus grammicus *Wiegmann 1828	Diarrhea	[103]

*Phrynosoma orbiculare *(Duméril & Bocourt 1870)	Unspecified	[91]

**Anguidae**		

*Abronia lythrochila *Smith & Alvarez Del Toro 1963	Unspecified	[38]

*Mesaspis moreletii *Bocourt 1871	Unspecified	[38]

**Tropiduridae**		

*Tropidurus hispidus *(Spix, 1825) - Lizard	Alcoholism, dermatomycosis, warts, abscesses, boils, sore throat, erysipelas, healing of umbilical cord of newborn baby	[7-9,31]

*Tropidurus semitaeniatus *(Spix, 1825) - Lizard	Measles, asthma, alcoholism, dermatomycosis, warts	[8,62,97,162]

*Tropidurus torquatus *(Wied, 1820) - Lizard	Chicken pox	[7-9,31,62,71,97,115,162]

*Uranoscodon superciliosus *(Linnaeus, 1758)	As a sedative	[8,62,69,131]

**Boidae**		

*Boa constrictor *(Linnaeus, 1758) - Boa	Rheumatism, lung disease, thrombosis, boils, tuberculosis, stomach ache, edema, snake bite, cancer, pains, swelling, to prevent abort, pain in the body, inflammation, athlete's foot, calluses, tumours, cracks in the sole of the feets, goiter, sore throat, arthrosis, insect sting, dog bite, erysipelas, asthma, neck strain, strain muscle, backache, contusions and muscular pain	[7-10,29,31,62,70,97,121,186,188]

*Corallus caninus *(Linnaeus, 1758) - American emerald tree boa	Pain relief caused by sting of animals	[8,29,62]

*Corallus hortolanus *(Linnaeus, 1758) - Snake	To assist in removing spines or other sharp structures from the skin, rheumatism	[8,29,62]

*Eunectes murinus *(Linnaeus, 1758) - Anaconda	Wounds, skin problems, bruises, sprains, arthrosis, rheumatism, boils, sexual impotence, headache, sore throat, thrombosis, swelling, tumour, asthma, muscle strain, numbness, syphilis, to reduce pain, luxation	[8,29,62,71]

*Eunectes notaeus *(Cope, 1862)- Sucuri	Unspecified	[160]

*Epicrates cenchria *(Linnaeus, 1758) - Brazilian rainbow boa	Rheumatism, pain in articulations, snake bite, sore throat	[8,29,62,71,97]

**Colubridae**		

*Leptophis ahetula *(Linnaeus, 1758) - Parrot snake	Pain relief caused by sting of animals	[8,29,62]

*Mastigodryas bifossatus *(Raddi, 1820) - Rio tropical racer	Snake bites	[8,29,62,108]

*Pituophis lineaticollis *Cope 1861	Post partum cold, pains corporeal	[40]

*Oxyrhopus trigeminus *Duméril, Bibron & Duméril, 1854	Rheumatism	[8,29,62,108]

*Oxyrhopus formosus *Wied 1820	Unspecified	[174]

*Oxyrhopus melanogenys *Tschudi 1845	Unspecified	[174]

*Spilotes pullatus *(Linnaeus, 1758) - Tiger snake	Insects bite, snake bite	[8,29,62,108]

*Tachymenis peruviana *Wiegmann, 1835	Pain in the bones, pain in kidneys and to treat inflamations, tootache and scare, fractures	[148,189]

*Drymobius margaritiferus *Schlegel 1837	Mail aire, pain in bones, rheumatism	[38,148]

*Chironius carinatus *(Linnaeus, 1758)	Infertility	[151]

*Chironius grandisquamis *Peters 1869	Unspecified	[174]

*Lampropeltis triangulum *Lacépède 1789	Unspecified	[38,148]

*Imantodes cenchoa *Linnaeus 1758	Unspecified	[174]

*Leptodeira annulata *(Linnaeus, 1758)	Unspecified	[174]

**Crotalidae**		

*Caudisona durissa *(Linnaeus, 1758) - Neotropical rattlesnake	Asthma, snake bite, thrombosis, wounds, luxation, rheumatism, pain in the legs, erysipelas, deafness, epilepsy, skin diseases, tuberculosis, hanseniasis, backache, tumour, boil, headache, earache, osteoporosis, sore throat, toothache, insects bite, irritation when milk teeth are erupting, cancer, to accelerate parturition, pain in the body, avoid pregnancy, mail aire, swellings, bone aches, gastritis, eczema	[8,29,62,63,71,97,108,139,161,164-166]

*Crotalus polystictus *Cope 1865	Unspecified	[125]

*Crotalus transversus *Taylor 1944	Unspecified	[125]

*Crotalus triseriatus *Wagler 1830	Unspecified	[125]

*Crotalus durissus *Linnaeus 1758	Wounds, disorders in parturition, lepra, cancer, acne	[38,105]

*Crotalus atrox *Baird & Girard 1853	Pneumonia, muscular pain, sight, sore throat, gangrene, varicose veins, ulcer	[103]

*Crotalus basiliscus *COPE 1864	Unspecified	[78]

**Elapidae**		

*Micrurus spixii *Wagler 1824	Unspecified	[174]

*Micrurus surinamensis *Cuvier 1817	Unspecified	[174]

*Micrurus ibiboboca *(Merrem, 1820)	Rheumatism, snake bite	[62,108,139]

**Viperidae**		

*Bothrops leucurus *Wagler, 1824 - Lance head	Tumour, boils	[62,108,139]

*Bothrops asper *Garman 1883	Unspecified	[38,148,152]

*Bothrops atrox *Linnaeus 1758	Unspecified	[80,185]

*Lachesis muta *(Linnaeus, 1766) - Bushmaster	Rheumatism, swelling, tumour, boil, insects bite, snake bite	[8]

*Cerrophidion tzotzilorum *Campbell 1985	Rheumatism, mail aire	[38,148]

*Atropoides nummifer *Rüppell 1845	Unspecified	[38,148]

**Dipsadidae**		

*Philodryas olfersii *Lichenstein, 1823	Unspecified	[80]

*Thamnodynastes strigatus *(Günther, 1858)	Snake bite	[151]

**Chelidae**		

*Chelus fimbriatus *(Schneider, 1783) - Matá-matá	Impotence	[82,87,153]

*Phrynops geoffroanus *(Schweigger, 1812) - Geoffroy's side-necked turtle	Asthma, sore throat, swelling, earache, rheumatism, arthrosis, healing of umbilical cord of newborn baby, mumps, ingrown nail, eczema, articulation problems, wounds	[7-9,71]

*Phrynops tuberosus *Peters 187	Asthma, rheumatism and bruises	[109]

*Mesoclemmys *tuberculata (Luederwaldt, 1926) - Tuberculate toadhead turtle	Rheumatism, discharge, thrombosis, bronchits, diarrhoea, haemorrhag, asthma, sore throat, hoarseness	[62,97,108,161]

*Mauremys leprosa *Schweigger, 1812	Unspecified	[80]

**Cheloniidae**		

*Caretta caretta *(Linnaeus, 1758) - Loggerhead turtle	Injuries caused by bang, toothache, diabetes, headache, backache, wounds, cough, bronchits, asthma, thrombosis, rheumatism, stroke, hoarseness, flu, backache, earache, sore throat, swelling	[7-9,62,139,142,162]

*Chelonia mydas *(Linnaeus, 1758) - Green sea turtle	Injuries caused by bang, toothache, diabetes, headache, backache, wounds, cough, bronchits, asthma, flu, thrombosis, rheumatism, stroke, hoarseness, earache, sore throat, swelling, whooping cough, arthritis, erysipelas, boil, arthrosis, inflammation	[7-9,62,68,139,142]

*Eretmochelys imbricata *(Linnaeus, 1766) - Atlantic hawksbill	Injuries caused by bang, toothache, diabetes, headache, backache, wounds, cough, bronchits, asthma, thrombosis, stroke, hoarseness, flu, rheumatism, earache, sore throat, swelling	[7-9,62,139,142,162]

*Lepidochelys olivacea *(Eschscholtz, 1829)	Injuries caused by bang, toothache, diabetes, headache, backache, wounds, cough, flu, bronchitis, asthma, thrombosis, rheumatism, stroke, hoarseness	[62,114,119,162]

**Dermochelyidae**		

*Dermochelys coriacea *(Vandelli, 1761) - Leatherback turtle	Rheumatism, earache, sore throat, swelling	[7-9,62,139,142]

**Geoemydidae**		

*Rhinoclemmys punctularia *(Daudin, 1802) - Spot-legged turtle	Wounds, tumour, erysipelas, earache, rheumatism	[7-9,62,139,142]

**Podocnemididae**		

*Podocnemis expansa *(Schweiger,1812) - Amazon river turtle	Inflammation, acne, tumour, boil, rheumatism, pterygium, skin spots, backache, earache, arthrosis, arthritis, swelling, wrinkle	[7-9,62,139,141]

*Podocnemis unifilis *(Troschel, 1848) - Yellow-spotted river turtle	Wounds, tumour, erysipelas, earache, rheumatism	[7-9,62,139,188]

*Podocnemis sextuberculata *Cornalia, 1849 - Six-tubercled Amazon River turtle	Blackhead, acne	[62,69]

*Peltocephalus dumeriliana *Schweigger 1812	Blackhead, acne	[62,69]

**Testudinidae**		

*Chelonoidis carbonaria *(Spix, 1824) - Red-footed tortoise	Catarrh, erysipelas, bronchitis, to stop the sensation to getting thirsty, asthma	[8,9,31,63,115,162,164]

*Chelonoidis denticulata *(Linnaeus, 1766) - Yellowfooted tortoise	Sore throat, rheumatism, hernia, wounds, leishmaniosis, varicocele, earache, part of woman's body, asthma, pains	[8,9,31,87,157]

*Gopherus flavomarginatus *Legler 1959	Arthritis	[103]

**Kinosternidae**		

*Staurotypus triporcatus *Wiegmann 1828	Unspecified	[93]

*Kinosternon scorpioides *Linnaeus 1766	Hermaphroditism, malaria, **t**uberculosis	[38,62]

*Kinosternon integrum *Le Conte 1854	Smallpox	[105]

**Alligatoridae**		

*Caiman crocodilus *(Linnaeus, 1758) - Common cayman	Asthma, stroke, bronchitis, backache, earache, rheumatism, thrombosis, sexual impotence, snake bites, evil eye, irritation when milk teeth are erupting, discharge, swelling, scratch, athlete's foot, ophthalmological problems, sore throat, amulet used as a protection against snake bite, hernia, prostate problems, infection, inflammation, epilepsy	[7-9,30,62,63,108,139,162-164,166,188]

*Caiman latirostris *(Daudin, 1801) - Cayman	Asthma, sore throat, amulet used as a protection against snake bite, rheumatism, irritation when milk teeth are erupting, hernia, prostate problems	[7-9,62,139]

*Caiman yacare *Daudin 1802	Unspecified	[180]

*Melanosuchus niger *(Spix, 1825) - Black cayman	Thrombosis, infection, swelling, asthma, amulet used as a protection against snake bite, injuries caused by spines of the 'arraia', pain relief in injuries caused by snake bites	[7-9,62,139]

*Paleosuchus palpebrosus *(Cuvier, 1807) - Cayman	Snake bite, asthma, stroke, rheumatism, thrombosis, backache, sexual impotence, edema, mycosis, evil eye, irritation when milk teeth are erupting, discharge, sore throat, amulet used as a protection against snake bite, hernia, prostate problems	[7-9,62,71,139]

*Paleosuchus trigonatus *(Schneider, 1801)	Rheumatism	[62,69]

**Crocodylidae**		

*Crocodylus moreletii *Duméril & Bibron 1851	Asthma, bronchial diseases	[93]

*Crocodylus acutus *Cuvier 1807	Unspecified	[152]

**BIRDS**		

**Turdidae**		

*Turdus chiguanco *Lafresnaye & d'Orbigny, 1837	Stomach ache	[189]

*Turdus grayi *Bonaparte, 1838	Unspecified	[152]

*Troglodytes musculus *Naumann, 1823	Epilepsy	[72]

**Cariamidae**		

*Cariama cristata *(Linnaeus, 1766)	Ethnoveterinary use	[127,178]

**Accipitridae**		

*Geranoaetus melanoleucus *(Vieillot, 1819) - Black-chested Buzzard-Eagle	The feathers are used as inensing and to makemasks, wrinkles.	[148]

*Harpia harpyja *(Linnaeus, 1758)	Unspecified	[179]

*Buteogallus urubitinga *(J. F. Gmelin, 1788)	Backache, column pain and rheumatism	[66]

*Spizastur melanoleucus *(Vieillot, 1816)	Unspecified	[152]

*Parabuteo unicinctus *(Temminck, 1824)	Snake bite	[152]

**Anatidae**		

*Anser anser *(Linnaeus, 1758) - Greylag goose	Laryngitis, pharyngitis, tonsillitis	[97]

*Anas platyrhynchos *Linnaeus, 1758 - mallard	General weaknesses, sexual weakness, nervous disturbances	[66]

*Netta erythrophthalma *(Wied-Neuwied, 1833)- 'paturi'	Male impotence and weakness	[66]

*Cairina moschata *(Linnaeus, 1758)	Unspecified	[111]

*Lophodytes cucullatus *(Linnaeus, 1758)	Unspecified	[96]

**Tytonidae**		

*Tyto alba *(Scopoli, 1769)	Unspecified	[152]

**Anhimidae**		

*Anhima cornuta *(Linnaeus, 1766) - Horned screamer, anuhma	Intoxication from poisonous animals	[151]

**Scolopacidae**		

*Actitis macularius *(Linnaeus, 1766)	Dandruff (seborrhea)	[96]

**Ardeidae**		

*Ardea cocoi *(Linnaeus, 1766) - White-necked Heron	Swelling, inflammation, injuries caused by the spines of the 'arraia' and others fishes, asthma, boil, tumour, rheumatism, earache	[7-9,87]

*Ardea alba *Linnaeus, 1758	Bronquithis and pneumonia	[148]

*Nycticorax nycticorax *(Linnaeus, 1758)	Unspecified	[96]

**Apodidae**		

*Streptoprocne zonaris *(Shaw, 1796)	Unspecified	[106]

*Casmerodius albus *(Gmelin, 1789) - Garça	Bronquithis and pneumonia	[71]

**Ardeidae**		

*Tigrisoma lineatum *(Boddaert, 1783) - socó, Rufescent Tiger-Heron	Bronquithis and pneumonia	[71]

**Caprimulgidae**		

*Nyctidromus albicollis *(Gmelin, 1789) - Pauraque	Amulets, snake bite	[69]

*Caprimulgus vociferus *A. Wilson, 1812	Unspecified	[38]

**Cathartidae**		

*Coragyps atratus *(Bechstein, 1793) - Black vulture	Deafness, bronchitis, anaemia, alcoholism, asthma, flu, earache, rheumatism, pain in the parturition, mal aire, swelling, epilepsy	[7-9,31,42,97]

*Cathartes aura *(Linnaeus, 1758) - Urubu, urubu-de-cabeça-vermelha	Unspecified	[4,91,103]

**Cracidae**		

*Penelope jacucaca *(Spix, 1825) - White-browed guan	Insomnia	[162]

*Penelope superciliaris *Temminck, 1815	Asthma	[177]

*Penelope purpurascens *Wagler, 1830	Unspecified	[152]

*Penelope jacquacu *Spix, 1825	Unspecified	[179]

*Crax globulosa *Spix, 1825 - Wattled Curassow	To cure rheumatism and to remove the "negative energy" from people	[184]

*Crax rubra *Linnaeus, 1758	Unspecified	[152]

*Aburria pipile *(Jacquin, 1784)	Unspecified	[179]

*Pauxi tuberosa *(Spix, 1825)- Razor-billed Curassow	Pneumonia, bleeding, children's lack of appetite, indigestion, stroke, insect" and snake bites	[74]

*Mitu tuberosum *(Spix, 1825)	Unspecified	[179]

*Ortalis guttata *(Spix, 1825)	Unspecified	[179]

*Ortalis vetula *(Wagler, 1830)	Rheumatism	[152]

**Psophiidae**		

*Psophia leucoptera *Spix, 1825	Unspecified	[179]

**Phoenicopteridae**		

*Phoenicopterus andinus *Philippi, 1854	To alliviate labor pain, sprains and distend	[148]

*Phoenicopterus chilensis *Molina, 1782	To alliviate labor pain, sprains and distend	[148]

*Phoenicopterus jamesi *Sclater, 1886	To alliviate labor pain, sprains and distend	[148]

**Ciconiidae**		

*Vultur gryphus *Linnaeus, 1758	Stomach ache, poor digestion, boils	[189]

*Ciconia maguari *(Gmelin, 1789) - Maguari stork	Injuries caused by the spines of the 'arraia' and others fishes, thrombosis	[7-9,82]

*Jabiru mycteria *(Lichtenstein, 1819) - Jabiru	Unspecified	[82]

*Sarcoramphus papa *(Linnaeus, 1758) - King Vulture	Epilepsia, sifilis, ulcera	[96]

**Columbidae**		

*Leptotila rufaxilla *(Richard & Bernard, 1792) - Gray-fronted dove	Thrombosis, pterygium	[66,162]

*Columba livia *(Gmelin, 1789) - Rock pigeon	Asthma, laryngitis, pharyngitis, tonsilitis	[66,97,103]

*Columba picazuro *Temminck, 1813 - 'asa-branca	Sore throat, tonsillitis, bronchitis and hoarseness	[66]

*Columbina talpacoti *(Temminck, 1810)	Unspecified	[175]

*Columbina passerina *(Linnaeus, 1758)	Unspecified	[96]

**Corvidae**		

*Cyanocorax cyanopogon *(Wied, 1821) - White-naped jay	Asthma, neurological problems	[66,81,162]

**Cotingidae**		

*Procnias nudicollis *(Vieillot, 1817) - araponga, Bare-throated Bellbird	Tuberculosis	[4,71]

*Cotinga amabilis *Gould, 1857	Unspecified	[96]

*Crotophaga ani *Linnaeus, 1758 - Smooth-billed ani	Bronchitis, thrombosis, asthma, whooping cough, rheumatism	[7-9,97]

*Crotophaga sulcirostris *Swainson, 1827	Cancer	[105]

*Piaya cayana *(Linnaeus, 1766)	Heart diseases	[105]

*Guira guira *(Gmelin, 1788) - Guira cuckoo	Asthma	[97]

*Geococcyx velox *(Wagner, 1836)	Unspecified	[91]

*Geococcyx californianus *(Lesson, 1829)	Cancer	[103]

**Charadriidae**		

*Vanellus chilensis *(Molina, 1782) - Southern lapwing	To stay awake	[4,115,162]

**Emberezidae**		

*Coereba flaveola *(Linnaeus, 1758)- Banana quit	Thrombosis	[162]

**Numididae**		

*Numida meleagris *Linnaeus, 1758 - Helmeted Guineafowl, "Guiné"	Whooping cough	[4]

**Falconidae**		

*Herpetotheres cachinnans *(Linnaeus, 1758) - Laughing falcon	Snake bite, sore throat, tonsillitis and hoarseness	[66,69]

*Caracara plancus *(Miller, 1777) - Southern caracara	Snake bite	[69]

*Falco rufigularis *Daudin, 1800 - Bat falcon	Snake bite	[69,82]

**Opisthocomidae**		

*Opisthocomus hoazin *(Statius Muller, 1776)	Unspecified	[82]

**Furnaridae**		

*Furnarius rufus *(Gmelin, 1788)- Rufous hornero	Mumps	[4,114]

**Meleagrididae**		

*Meleagris gallopavo *Linnaeus, 1758 - turkey	Asthma	[7,9]

**Odontophoridae**		

*Odontophorus capueira *(Spix, 1825)	Unspecified	[111]

*Callipepla squamata *(Vigors, 1830)	Unspecified	[96]

*Cyrtonyx montezumae *(Vigors, 1830)	Evil eye	[103]

*Colinus virginianus *(Linnaeus, 1758)	Dandruff, fever	[96]

**Phasianidae**		

*Gallus gallus *(Linnaeus, 1758) - Domestic chicken	Catarrh, fever, warts, haemorrhage, bronchitis, nasal congestion, flu, skin thorns and wounds, asthma, sore throat, tumour, poor digestion, healing of umbilical cord of newborn baby, swelling, cough, tuberculosis, earache, tonsillitis, rheumatism, diarrhoea, inflammation, pneumonia, Renal calculus, headache, sore throat, nasal congestion, fever, swelling	[7-9,31,42,63,68,81,85,97,103,109,110,115,162,164,166]

*Pavo cristatus *Linnaeus, 1758 - Indian peafowl	Thrombosis, epilepsy, fatigue	[30,126]

*Coturnix coturnix *(Linnaeus, 1758)-'codorna	Male impotence, urinary infection and weakness	[66]

**Picidae**		

*Dryocopus lineatus *(Linnaeus, 1766) - Lineated woodpecker	Sexual impotence	[69]

*Colaptes rupicola *Orbigny, 1840	Unspecified	[189]

*Melanerpes formicivorus *(Swainson, 1827)	Epilepsy	[105]

*Melanerpes aurifrons *(Wagler, 1829)	Headache	[96]

**Psittacidae**		

*Amazona aestiva *(Linnaeus, 1758)	Asthma	[7,9]

*Ara ambiguus *(Bechstein, 1811)	Unspecified	[152]

*Amazona farinosa *(Boddaert, 1783)	Unspecified	[152]

*Amazona autumnalis *(Linnaeus, 1758)	Unspecified	[152]

*Amazona auropalliata *(Lesson, 1842)	Unspecified	[152]

*Amazona albifrons *(Sparrman, 1788)	Unspecified	[152]

*Pionus senilis *(Spix, 1824)	Unspecified	[152]

*Pionus menstruus *(Linnaeus, 1766)	Unspecified	[152]

*Pionopsitta haematotis *(P. L. Sclater & Salvin, 1860)	Unspecified	[152]

*Ara macao *(Linnaeus, 1758)	Mental problems	[152]

**Hirundinidae**		

*Petrochelidon andecola *(D'Orbigny & Lafresnaye, 1837)	Epilepsy, heart diseases	[189]

*Stelgidopteryx ruficollis *(Vieillot, 1817)	Cyst	[175]

*Hirundo rustica *Linnaeus, 1758 - Barn Swallow	Dandruff, muscular pain, ophthalmological problems, Insomnia,	[96]

**Strigidae**		

*Glaucidium brasilianum *(Gmelin, 1788)	Rheumatism	[152]

**Alcedinidae**		

*Chloroceryle americana *(Gmelin, 1788)	Unspecified	[152]

**Cardinalidae**		

*Passerina versicolor *(Bonaparte, 1838)	Unspecified	[96]

**Rallidae**		

*Aramides cajanea *(Statius Muller, 1776) - Grey-necked wood-rail	Evil eye	[162]

**Struthionidae**		

*Struthio camelus *Linnaeus, 1766 - Common Ostrich	Osteoporosis	[136]

**Ramphastidae**		

*Ramphastos tucanus *Linnaeus, 1758 - Red-billed Toucan	Thrombosis, sexual impotence	[31,69]

*Ramphastos vitellinus *Lichtenstein, 1823 - Channel-billed toucan	Sexual impotence	[69]

*Ramphastos sulfuratus *Lesson, 1830	Unspecified	[77]

*Pteroglossus aracari *(Linnaeus, 1758) - Black-necked aracari	Sexual impotence	[69]

*Pteroglossus inscriptus *- Swainson, 1822, Lettered aracari	Sexual impotence	[69]

*Pteroglossus torquatus *(Gmelin, 1788)	Unspecified	[152]

*Pteroglossus frantzii *Cabanis, 1861	Unspecified	[152]

*Selenidera spectabilis *Cassin, 1858	Unspecified	[152]

**Rheidae**		

*Rhea americana *(Linnaeus, 1758) - Greater rhea	General aches, rheumatism, thrombosis, strokes	[63,115,162,164,166]

*Pterocnemia pennata *(Orbigny, 1834)	Unspecified	[148]

**Trogonidae**		

*Pharomachrus mocinno *De la Llave, 1832	Unspecified	[152]

**Tinamidae**		

*Crypturellus noctivagus *(Wied, 1820) - Yellow-legged tinamou	Thrombosis, stroke, snake bites, tuberculosis, deafness	[71,115,162]

*Crypturellus variegatus *(Gmelin, 1789) - Inambu-galinha	Unspecified	[87]

*Nothura boraquira *(Spix, 1825) - White-bellied nothura	Thrombosis, stroke, tootache	[115,162]

*Nothura maculosa cearensis *Naumburg, 1932 - Spotted Nothura	Effusion, snake bite	[178]

*Rhynchotus rufescens *(Temminck, 1815) - Red-winged tinamou	Snake bite, thrombosis, tuberculosis	[119,162]

*Tinamus solitarius *(Viellot 1819) - Macuco, macuca	Unspecified	[111]

**Trochilidae**		

*Eupetomena macroura *(Gmelin, 1788) - Swallow-tailed hummingbird	Cardiopathies, asthma, flu, pains	[97]

*Lesbia nuna *(Lesson, 1832)	Evil eye	[189]

**Tyrannidae**		

*Fluvicola nengeta *(Linnaeus, 1766) - Masked water-tyrant	Boils, asthma	[66]

*Pitangus sulphuratus *(Linnaeus, 1766)	Earache	[97]

**Icteridae**		

*Quiscalus mexicanus *(Gmelin, 1788)	Unspecified	[38]

*Psarocolius wagleri *(G. R. Gray, 1845)	Unspecified	[152]

**Emberizidae**		

*Zonotrichia capensis *(Statius Muller, 1776)	Unspecified	[189]

**MAMMALS**		

**Molossidae**		

*Molossus molossus *(Pallas, 1766), Pallas' free-tailed bat (Bat)	Asthma	[66,176]

**Noctilionidae**		

*Noctilio leporinus *(Linnaeus, 1758) - greater bulldog bat	Unspecified	[152]

**Phyllostomidae**		

*Artibeus jamaicensis *Leach, 1821 - bat	Cyst	[175]

**Agoutidae**		

*Agouti paca *(Linnaeus, 1766) - Spotted paca	Wound in the breast caused by suckling, ophthalmological problems, stomach disorders, pterygium, to suck a splinter out of skin or flesh, injuries caused by the spines of 'arraia', control cholesterol level, thrombosis, general body pain, leishmaniasis, snake bite, rheumatism, heart pain, pain in bones, liver pain, fever, child birth, ant bite	[7-9,31,70,85,97,162]

**Balaenopteridae**		

*Megaptera novaeangliae *Borowski, 1781	Unspecified	[80,171]

*Balaenoptera acutorostrata *Lacépède, 1804 - Minke whale	Rheumatism, sore throat, wounds	[7-9]

**Bovidae**		

*Bos taurus *Linnaeus, 1758 - Cow	Thrombosis, evil eye, amulet used as a protection against snake bite, baldness, sexual impotence, measles, varicella, anaemia, whooping cough, Alcoholism, rheumatism, inflammation, asthma, cough, sore throat, wounds, cracks in the sole of the feet, bronchitis, dizziness, bladder problems, varicella, removal of thorns, paralisia facial, nervousness, earache, migraine,	[7-9,31,36,42,63,115,162-164,166]

*Bubalus bubalis *(Linnaeus, 1758) - Water buffalo (feral)	Rheumatism, osteoporosis, thrombosis	[8]

*Ovis aries *(Linnaeus, 1758) - Sheep	Edema, fractures, erysipelas, herpes zoster, backache, swelling, to assist children who take longer than usual to start walking, arthritis, arthrosis, rheumatism, muscle strain, inflammation, luxation, cracks in the sole of the feet, joint pain, removal of thorns	[7-9,31,36,42,63,91,97,115,162-164,166]

*Capra hircus *Linnaeus, 1758 - Domestic goat	Evil eye, snake bite, muscle strain	[7-9,31,42,91]

**Geomyidae**		

*Orthogeomys hispidus *(LeConte, 1852)	Stomach ache, fever, *susto *and *espanto*, swelling, madness	[38,40]

**Bradypodidae**		

*Bradypus variegatus *Shinz, 1825 - Brown-throated three-toed sloth	Thrombosis	[8,30,97]

*Bradypus tridactylus *Linnaeus, 1758 - Pale-throated three-toed sloth	Thrombosis, insects bite, scorpions bite	[70]

**Canidae**		

*Lycalopex culpaeus *(Molina, 1782)	Scare, susto'' (fright)	[154]

*Lycalopex gymnocercus *(G. Fischer, 1814)	Air loss, asthma, backache, disorders after parturition, pain in bones, rheumatism, scare, sprains, ulcer	[100,154]

*Canis lupus *(Linnaeus, 1758) - Domestic dog	Chicken pox, mumps, smallpox, asthma, varicella, measles, menstrual cramps	[7-9,31,97,115,162]

*Canis latrans *Say, 1823	Rheumatism	[8,11,91,103]

*Cerdocyon thous *(Linnaeus, 1766) - Crab-eating fox	Rheumatism, flu, haemorrhoids, disorders after parturition, diabetes, thrombosis, backache, osteoporosis, eczema, pain in articulations, sore throat, womb inflammation	[7-9,31,42,134]

*Chrysocyon brachyurus *(Illiger, 1815) - Maned wolf	Epilepsy	[133,161,162]

*Dusicyon thous *- Linnaeus, 1766 - Crab-eating fox	Alcoholism, thrombosis, rheumatism, ophthalmological problems, diabetes, urinary infection	[97,133]

*Speothos venaticus *(Lund, 1842) - Bush dog	Haemorrhoids	[97,133]

**Caviidae**		

*Galea spixii *Wagler, 1831	Unspecified	[80]

*Cavia aperea *Erxleben, 1777	Inflammation, teething	[97]

*Cavia porcellus *(Linnaeus, 1758)	nervios encogidos.	[182]

*Kerodon rupestris *(Wied-Neuwied, 1820)	Constipation, tired sight, effusion	[162]

**Cebidae**		

*Alouatta belzebul *(Linnaeus, 1766) - Red-handed howler monkey	Whooping cough, sore throat, asthma	[7-9,137]

*Alouatta *nigerrima Lönnberg, 1941 - Amazon black howler	Whooping cough, inflammation	[69,137,181]

*Alouatta seniculus *(Linnaeus, 1766) - Red howler monkey	Whooping cough, inflammation, to accelerate parturition	[69,87,137]

*Allouatta fusca *(É. Geoffroy, 1812) - *Bugio*	Used as aphrodisiac; used to treat any disease	[101,111]

*Alouatta sara Elliot, 1910*	Unspecified	[83,137]

*Alouatta palliata *(Gray 1849)	General pains and inflammation	[107,137]

*Ateles chamek *(Humboldt, 1812)	Fever, cough, cold shoulder pain, sleeping problems, leishmaniosis, spider bite, snake bites.	[83,137]

*Ateles geoffroyi *Kuhl, 1820	Rheumatism	[137,152]

*Ateles paniscus *(Linnaeus 1758)	Rheumatism	[137]

*Aotus azarai *(Humboldt, 1811)	To cure dribbling in babies	[137]

*Aotus griseimembra *Elliot 1912	Unspecified	[137]

*Cebus apella *(Linnaeus, 1758) - Brow capuchin	Insect sting, eye infection, Inflammatory processes, insect sting, used for osteomuscular problems, eye infection and male impotency	[7-9,42,137]

*Cebus albifrons *(Humboldt 1812	Used as fortifier	[95,137,152]

*Cebus capucinus *(Linnaeus 1758)	Unspecified	[137]

*Lagothrix lagotricha *(Humboldt 1812)	Unspecified	[95,137]

**Callitrichidae**		

*Saguinus fuscicollis *(Spix, 1823)	Impotence	[83]

*Saguinus mystax *(Spix 1823)	Impotence	[83]

**Cervidae**		

*Blastocerus dichotomus *(Illiger, 1815) - Marsh deer	Diarrhoea, vomit	[151]

*Mazama americana *(Erxleben, 1777) - Red brocket	Stroke, cold, burns	[7-9,30,42,115]

*Mazama simplicicornis *(Illinger, 1811)	Diarrhoea, verminosis, evil eye	[69]

*Mazama *cf. *gouazoupira *(G. Fischer, 1814) - Gray brocket	Asthma, edema, rheumatism, snake bite, thrombosis, to assit children who take longer than usual to start walking, tootache, wounds, sprains	[8,63,97,162,164]

*Ozotocerus bezoarticus *(Linnaeus, 1758)	Diarrhoea, verminosis, evil eye	[69])

*Odocoileus virginianus *(Zimmermann, 1780)	heart diseases, oftalmological problems, Didelphis virginiana	[91,103]

**Dasypodidae**		

*Dasypus novemcinctus *(Linnaeus, 1758) - Nine-banded armadillo	Thrombosis, insects bite, scorpions bite, edema, asthma, deafness, earache, evil eye, diarrhoea, whooping cough, tuberculosis, to accelerate parturition	[7,8,31,42,97,162]

*Dasypus kappleri *Krauss, 1862	Earache	[84]

*Euphractus sexcinctus *(Linnaeus, 1758) - Six-banded armadillo	Wounds, earache, evil eye, asthma, sore throat, pneumonia, sinusitis, deafness, coarse throat	[7,8,31,42,97,162]

*Tolypeutes tricinctus *(Linnaeus, 1758) - Brazilian three-banded armadillo	Thrombosis, rheumatism	[151,162]

*Priodontes maximus *(Kerr, 1792)	Embolism, ant bite, visions, skin diseases	[148]

*Chaetophractus vellerosus *(Gray, 1865)	General diseases	[148]

*Cabassous unicinctus *(Linnaeus, 1758) - Tatu-rabo-de-couro	Unspecified	[88,90]

*Cabassous centralis *(Miller, 1899) - Northern Naked-tailed Armadillo	Stomach ache	[104,152]

**Dasyproctidae**		

*Dasyprocta prymnolopha *Wagler, 1831 - Black-rumped agouti	Asthma, thrombosis	[4,162]

*Dasyprocta variegata - *Brown agouti	Childbirth, Impotence, snake bite	[148]

**Delphinidae**		

*Sotalia fluviatilis *Gervais & Deville, 1853) - Gray dolphin, gray river dolphin	Asthma, headache, rheumatism, hernia, womb disorders, sore throat, injuries caused by the spines of the 'arraia', swelling, haemorrhoids inflammation, wounds, earache, erysipelas, athlete's foot, tumour, cancer	[7-9,31,42,143]

*Sotalia guianensis *(P. J. Van Bénéden, 1864) - Guianan river dolphin	Asthma, headache, rheumatism, hernia, womb disorders, sore throat, injuries caused by the spines of the 'arraia', swelling, haemorrhoids inflammation, wounds, earache, erysipelas, athlete's foot, tumour, cancer	[7-9,31,42,143]

**Didelphidae**		

*Didelphis albiventris *(Lund, 1840) - Common opossum	Boils, rheumatism	[8]

*Didelphis marsupialis *(Linnaeus, 1758) - Southern opossum	Acne, wounds, bronchitis, joint pain, stomach ache, rheumatism, diarrhoea, inflammation, erysipelas, pain in gestation, asthma, headache, oothache, earache, sore throat, flu, fever, body pain, fatigue, to accelerate parturition, mal aire, swelling	[8,9,31,42,85]

*Didelphis virginiana *Kerr, 1792	Rheumatism, skin spots, acne, anaemia, to accelerate parturition, mal aire, swelling	[91,103]

*Didelphis aurita *(Wied-Neuwied, 1826) - Saruê	Unspecified	[88,90]

*Philander opossum *(Linnaeus, 1758) - Gray Four-eyed Opossum	Unspecified	[152]

**Megalonychidae**		

*Choloepus hoffmanni *Peters, 1858	Visions, hallucination, cramps	[148]

**Erethizontidae**		

*Coendou bicolor (Tschudi, 1844)*	Hallucination, fever, ant bite, flu, whooping cough, scare, varicose veins	[30]

*Coendou prehensilis *(Linnaeus, 1758) - Brazilian porcupine	Bronchitis, thrombosis, epilepsy, stroke, abscesses, conjunctivitis, asthma	[7-9,36,42,63,73,97,115,161,162,164]

*Coendou villosus *(Cuvier, 1822) - Ouriço-cacheiro	Unspecified	[111]

*Sphiggurus mexicanus *(Kerr, 1792)	Acne, wart	[91]

*Sphiggurus insidiosus *(Lichtenstein, 1818) - Luís-cacheiro	Unspecified	[89]

*Chaetomys subspinosus*(Olfers, 1818) - Luís-cacheiro	Unspecified	[89]

**Equidae**		

*Equus asinus *Linnaeus, 1758 - Asino	Snake bite, whooping cough, asthma, Avoid pregnancy	[7-9,42,73,91,115,162]

*Equus caballus *(Linnaeus, 1758) - Horse	Cough, deep cuts, dermatosis, wounds	[8]

**Felidae**		

*Felis silvestris *Schreber, 1775 - Domestic cat	Asthma, snake bites	[8]

*Puma concolor *(Linnaeus, 1771) - Mountain lion	Wounds, leishmaniosis, arthritis, pain in bones, rheumatism, distend, scare, stomach ache, evil eye, fever, avoid acne, contusions and muscular pain	[8,87,157]

*Panthera onca *(Linnaeus, 1758)	Wounds, leishmaniosis, Cough, fatigue, fever, pain in bones	[8,87,157]

*Panthera tigris *(Linnaeus, 1758) - Tigre	Unspecified	[4]

*Leopardus jacobitus *(Cornalia, 1865)	Self encorage	[148]

*Leopardus colocolo *(Molina, 1782)	Self encorage	[148]

*Leopardus wiedii *(Schinz, 1821)	Unspecified	[148]

*Leopardus pardalis *(Linnaeus, 1758) - Gato-maracajá	Unspecified	[30]

**Octodontidae**		

*Ctenomys opimus *Wagner, 1848	To make the child's teeth stronger	[148]

**Chinchillidae**		

*Lagidium viscacia *(Molina, 1782)	Bad memory	[148]

*Lagidium peruanum *Meyen, 1833	Earache	[182,189]

**Hydrochaeridae**		

*Hydrochaeris hydrochaeris *(Linnaeus, 1766) - Capybara	Thrombosis, conjunctivitis, venereal disease, rheumatism, earache, strengthen bones, liver pain, bronchitis, asthma, wounds, erysipelas, cough	[7-9,42,73,85,97,115,155,161,162]

**Heteromyidae**		

*Heteromys desmarestianus *Gray, 1868	Unspecified	[38,148]

**Iniidae**		

*Inia geoffrensis *(Blainville, 1817) - Amazon river dolphin	Asthma, headache, rheumatism, hernia, womb disorders, sore throat, injuries caused by the spines of the 'arraia', swelling, haemorrhoids inflammation, wounds, earache, erysipelas, athlete's foot, tumour, cancer	[7,8,42,73,143]

**Leporidae**		

*Sylvilagus brasiliensis *(Linnaeus, 1758) - Forest rabbit, tapeti	Thrombosis, conjunctivitis, boils, burns, ophthalmological problems, embolism, scare, fever, hallucination	[7,8,42,73,97,143,162]

*Sylvilagus floridanus *(J. A. Allen, 1890)	Unspecified	[152]

*Sylvilagus cunicularius *(Waterhouse, 1848)	To bring good luck	[103]

*Oryctolagus cuniculus *(Linnaeus, 1758) - Coelho	Unspecified	[4]

*Lepus alleni *Mearns, 1890	Stomach ache	[103]

**Sciuridae**		

*Sciurus spadiceus *Olfers, 1818	Unspecified	[125]

*Sciurus deppei *Peters, 1863	Unspecified	[125]

*Sciurus aureogaster *F. Cuvier, 1829	Unspecified	[125]

*Ammospermophilus interpres *(Merriam, 1890	Inflammation	[103]

**Mephitidae**		

*Conepatus semistriatus *(Boddaert, 1785) - Striped hog-nosed skunk	Rheumatism, acne, scabies, blood problems, bronchial diseases, skin problems, asthma, nervous disturbances	[7-9,42,73,162]

*Conepatus chinga *(Molina, 1782) - Gambá, Molina's Hog-nosed Skunk	Thrombosis, rheumatism, general diseases	

*Conepatus leuconotus leuconotus *(Lichtenstein, 1832)	Blood disorders, acne, stomach ache, mal aire, swelling, undescended testicles, rabies, whooping cough, bone pain Acné, dolor muscular	[105]

*Mephitis macroura *Lichtenstein, 1832	Stomach ache, mal aire, swelling, undescended testicles, Rabies, whooping cough, pain in bone, asthma	[8,103,125,148]

*Spilogale putorius *(Linnaeus, 1758)	Stomach ache, mal aire, swelling, undescended testicles, rabies, whooping cough, pain in bone	[91]

**Ambystomidae**		

*Ambystoma mexicanum *(Shaw and Nodder, 1798)	Bronchitis	[103]

**Mustelidae**		

*Lontra longicaudis *(Olfers, 1818)	Thrombosis, ampollas	[162]

*Mustela frenata *Lichtenstein, 1831	Unspecified	[152]

*Eira barbara *(Linnaeus, 1758)	Unspecified	[152]

*Taxidea taxus *(Schreber, 1777)	Witchcraft	[103]

**Camelidae**		

*Lama glama *(Linnaeus, 1758)	Unspecified	[148,154]

*Lama guanicoe *(Müller, 1776)	Asthma, scare	[148,154]

*Vicugna vicugna *(Molina, 1782)	Unspecified	[148,154]

**Cyclopedidae**		

*Cyclopes didactylus *(Linnaeus, 1758)	Rheumatism	[152]

**Myrmecophagidae**		

*Myrmecophaga tridactyla *Linnaeus, 1758 - Giant anteater	Thrombosis, stroke, general body pain, Snake bite, urinary problem, heart pain, ant bite	[8,115,162]

*Myrmecophaga tetradactyla *(Linnaeus, 1758) - Collared anteater	Edema, thrombosis, itching, ant bite, rheumatism	[7-9,42,97]

*Tamandua mexicana *(Saussure, 1860) - Northern Tamandua	Unspecified	[152]

**Procyonidae**		

*Nasua nasua *(Linnaeus, 1766) - South American coati	Sexual impotence, wounds, skin burns, snake bites, backache, cold, cough, leg pain, wounded foot, earache, neck strain, to help become pregnant, whooping cough	[7-9,42,63,73,111,162,164,166]

*Nasua narica *(Linnaeus, 1766) White-nosed Coati English	Male impotence	[152]

*Procyon cancrivorus *(G. [Baron] Cuvier, 1798) - Crab-eating raccoon	Rheumatism, epilepsy, thrombosis, snake bite	[7-9,42,73,97,115]

*Procyon lotor *(Linnaeus, 1758)	Unspecified	[152]

*Potos flavus *(Schreber, 1774) - Kinkajou	Earache, snake bite, ant bite	[152]

**Physeteridae**		

*Physeter catodon *Linnaeus, 1758 - Sperm whale, cachelot	Asthma, backache, rheumatism, sore throat, wounds	[7-9,42,119]

**Muridae**		

*Neotoma mexicana *Baird, 1855	Unspecified	[38]

*Peromyscus mexicanus *(Saussure, 1860)	Abdominal distension	[40]

*Mus musculus *Linnaeus, 1758	Pertussis	[175]

**Suidae**		

*Sus scrofa *(Linnaeus, 1758) - Wild boar	Acne, boils, tumours, asthma, athlete's foot, warble, wounds,	[7-9,42,73,91]

**Tapiridae**		

*Tapirus terrestris *(Linnaeus, 1758) - Brazilian tapir	Rheumatism, arthrosis, osteoporosis, bursite, muscular pain, asthma, tonsillitis, cough, general body pain	[8]

*Tapirus bairdii *(Gill, 1865)	Unspecified	[152]

**Tayassuidae**		

*Pecari tajacu *Linnaeus 1758 - Collared peccary	Thrombosis, bronchitis, stroke	[8,85,115,162]

*Tayassu pecari *(Link, 1795) - White-lipped peccary	Thrombosis, stroke, cold, wounds	[8,9,115,162]

**Trichechidae**		

*Trichechus inunguis *(Natterer, 1883) - Amazonian manatee	Sprains, vaginal discharge, injuries caused by bang, burns, asthma, menstrual cramps, rheumatism, sore throat, wounds, muscle strain, suck a splinter out of skin or fresh, tumour, backache, hernia, arthrosis, luxation, insects bite	[7-9,31,42,155]

*Trichechus manatus *(Linnaeus, 1758) - Manatee	Arthrosis, luxation, menstrual cramps, insects bites, sprains, vaginal discharge, injuries caused by bang, burns, asthma, rheumatism, sore throat, wounds, muscle strain	[7-9,31,42,155]


**Figure 1:**
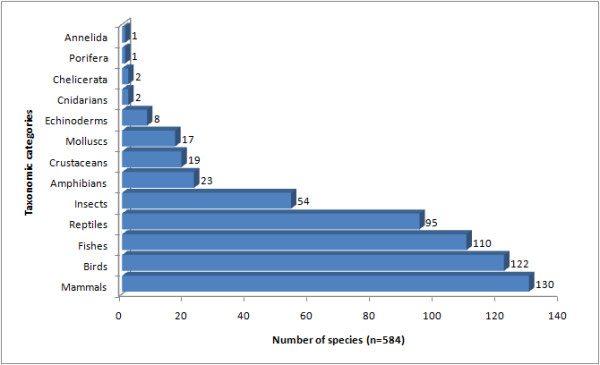
**Number of animal species used as remedies per taxonomic category in Latin America**.

**Figure 2:**
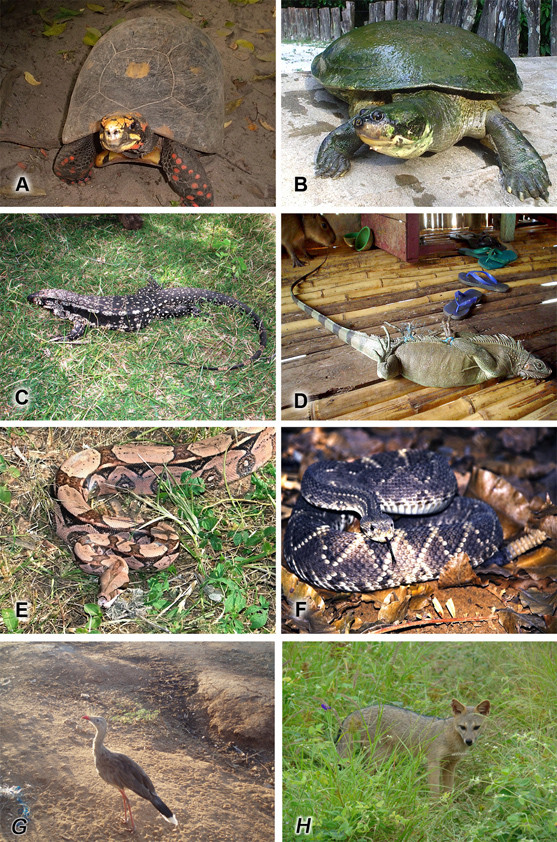
**Examples of animals used as medicine in Latin America**. A: *Chelonoidis carbonaria*, B: *Podocnemis expansa*, C *- Tupinambis merianae*, D - *Iguana iguana*, E - *Boa constrictor*, F - *Caudisona durissa*, G - *Cariama cristata*, H *- Cerdocyon thous*.

The species catalogued comprised 13 taxonomic categories, belonging to 215 families. The groups with the largest numbers of medicinal species were: mammals (with 130 species), followed by birds (122), fishes (110), reptiles (95) and insects (54) (Figure 1). Most medicinal animals recorded are vertebrates. Species of this group are also used frequently at countries of Europe, Africa and Asia countries[16,27,33,34,37,39,195-197]. Examples of animals used as medicine in Latin America is shown in Figure 2.

Most animals used as medicine were native to the Latin America, with the exception of *Hippocampus spinosissimus, H. trimaculatus *and *Panthera leo *and domestic exotic species (eg. *Bos taurus, Capra hircus *and *Ovis aries*). This finding demonstrates the importance of local biodiversity in furnishing folk medicines, in agreement with previous studies [7,8,42] which observed that faunal composition, accessibility, and availability directly influence the types of zootherapeutic items used in any given region. The use of the local fauna generally reduces the acquisition costs of commercial agents, and our results are in agreement with Apaza et al. [32], who noted a reduction in the cost of acquiring animal products in regions with abundant faunal resources. The medicinal use of wild exotic animals in Latin America indicates existence of international trade routes for medicinal species, a situation previously reported by Convention on International Trade in Endangered Species of Wild Fauna and Flora - CITES [198]. One excellent example are the seahorses. According to Baum and Vincent [79], the largest market for dried seahorses in Central America was for use as Traditional Chinese Medicine. These authors reported that three retailers in Panama City's Chinatown imported an estimated total of 18-27 kg dried seahorses annually from Hong Kong. These included *H. spinosissimus *and *H. trimaculatus*, which are found in the Red Sea and Indian Ocean, South-east Asia and Australia.

Some widespread species are used in different countries, such as *Tupinambis *spp. and *Boa constrictor *(in Argentina and Brazil), *Tapirus terrestris *(in Brazil and Bolivia) and *Caudisona durissa *in Mexico and Brazil [8,32,38,189]. Despite the fact that technical studies recording the use of animals in traditional medicine are all relatively recent, an analysis of historical documents and archaeological research indicated that animals have been used in traditional medicines since ancient times in Latin America [36,99,152-154,199]. In Brazil, for example, animal species have been used medicinally by indigenous societies for millennia. During his voyage through the interior of Brazil in the nineteenth century, Von Martius [200] recorded many natural medicines used by the Amerindian tribes he encountered, such as fresh caiman fat applied to alleviate rheumatism [115]. Even today, caiman fat (*Cayman latirostris, Melanosuchus niger *and *Paleosuchus palpebrosus*) is used in rural and urban communities to treat rheumatism [8]. Some examples of animals that have been used in Brazil since colonial times include: *Iguana iguana *(Iguanidae), *Caiman latirostris*, *Crotalus durissus*, and *Micrurus ibiboboca. *Similarly, a historical revision of the therapeutic uses of animals as described in Colonial chronicles from Mexico [99] revealed a total of 27 birds used as medicinal resource, showing their importance on this issue for the Ancient Mexican people.

## Illnesses and remedies

Animal-derived remedies were used for treating various diseases (See Table 1). A single illness could be treated by various animal species (e.g., 215 animal species were used in the treatment of asthma and 95 in the treatment of rheumatism), and many species were prescribed for treating multiple illnesses, as in the case of the products obtained from the teju (*Tupinambis teguixin*) and the snake boa (*Boa constrictor*), which were indicated to treat 29 and 30 conditions, respectively. The reported multiple therapeutic actions and the use of various animals for the same condition presumes different properties either of parts used or of modes of preparation, corroborating the remark by Iwu [201] that different chemical constituents are enhanced by different factors, such as preparation, dosage, or part used. Additionally, the possibility of using various remedies for the same ailment is popular because it permits adapting to the availability of the animals [7]. The fact that some medicinal animals are being used for the same purpose suggest that different species can share similar medicinal properties and might indicate the pharmacological effectiveness of these zootherapeutic remedies.

**Figure 3:**
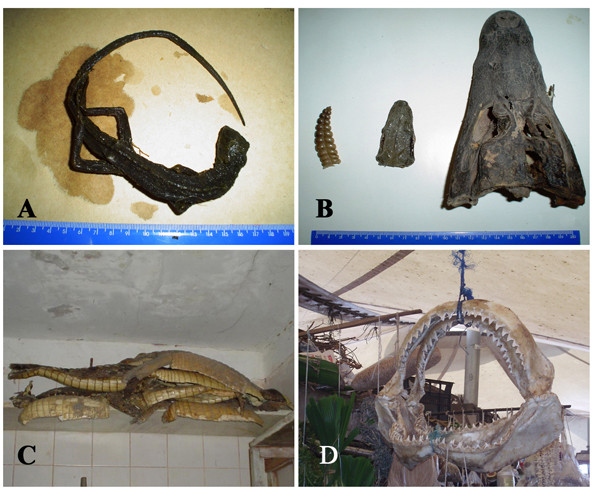
**Examples of animal products used as remedies in Latin America (Brazil)**. A - Tamanquaré (*Uranoscodon superciliosus*), B - Rattle of rattlesnake (*Caudisona durissa*), boas' head (*Boa constrictor*) and cayman's head (*Cayman *sp), C - Cayman's skin (*Cayman *sp.) and D - Shark jaws and teeth.

**Figure 4:**
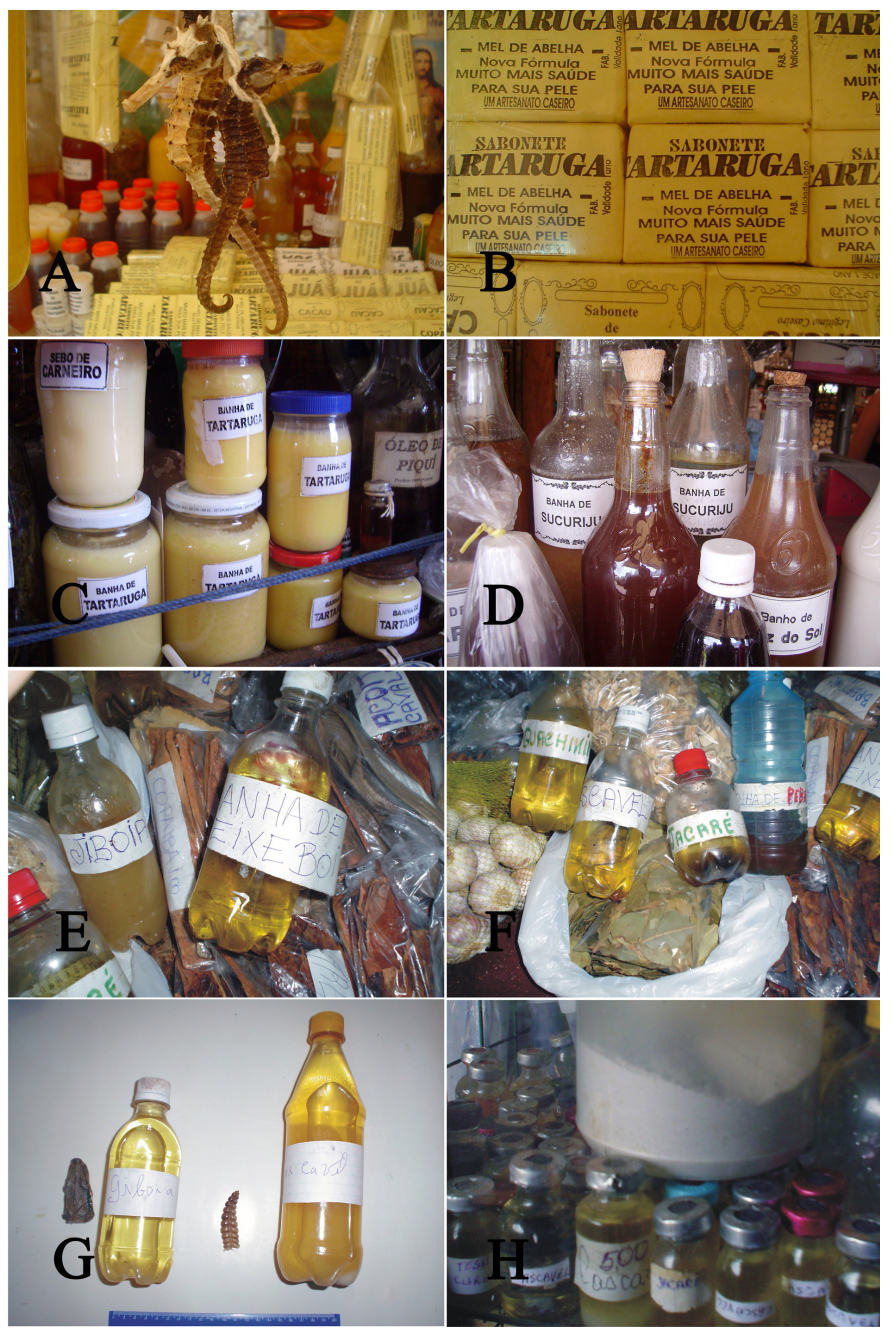
**Medicinal animal products sold in Brazilian cities**. A - Dried seahorses, B - Soap is produced from fat of turtle *P. expansa *and honey bee, C - Fat derived from sheep (*Ovis aries*) and Turtle (*P. expansa)*, D - Anaconda fat *(Eunectes murinus), E - *Boa fats (*Boa constrictor*) and manatee (*Trichecus *sp.), F - Plastic bottles with raccoon fat (*Procyon cancrivorus*), rattlesnake (*Caudisona durissa*), caymans (*Paleosuchus palpebrosus *or *Cayman crocodilus*) and armadillo (*Euphractus sexcintus*), G - Head and fat of boa (*B. constrictor*) and rattle and fat of rattlesnake (*C. durissa*), and H: Oyster powder (*Crassostrea rhizophorae*), fats of different animals prompt to be commercialized right in big pots of plastic and in small flasks.

Ingredients reportedly used in the preparation were fat, flesh, bone, bone marrow, cartilage, skin, tail, feather, liver, bile ("fel"), milk, rattle (from rattlesnakes), spine, shell, honey, wax, scale, rostral expansion, otolith, penis, carapace, blood, gizzard, beak, cocoon of insects, teeth, tongue, egg, egg shells, tibia, secretions, head, heart, urine, foot, legs, nest, guts, bezoar, ear, paw, spawn, nails, horn, sucking dish, eye, or more rarely, whole animals (Figures 3 and 4). Zootherapeutic products are consumed in several ways. Hard parts, such as teeth, nails, shells, rattles from snakes, fish scales, bone and cartilage generally are sun-dried, grated and crushed to powder, being then administered as tea or taken during meals, while fat, body secretion and oil are either ingested or used as an ointment. The influence of Westernization was also reflected in the presentation of some zootherapeutic products, which were either manufactured or pre-packaged. Examples are the fat extracted from the manatee (*Trichecus *sp.), sold as tablets, and the fat of Amazon River turtle (*Podocnemis expansa *(Schweiger, 1812)--Podocnemididae) sold as a manufactured soap in Brazil [42].

In many cases, the therapeutic use of animal remedies appears to be based on morphological or behavioral peculiarities of the animal in question. In México, for example, a tea made from the toasted and ground penis of the coatimundi (*Nasua narica *or *N. nasua*) is considered the most potent remedy for male impotence [8,40]. Descola [202] has recorded a similar remedy among the Achuar Jivaro of the Ecuadorian Amazon and provides an enlightening account of its probable origin: "The penis of the coati rejoices in a long fine bone that keeps it constantly rigid. This anatomical peculiarity has made a forceful impression upon the imagination of the Indians, and the men make the most of it, grating the bone into a decoction of green tobacco to make a love philtre. Quaffed at the right moment, it is reputed to prevent any flagging of the male member." In Brazil, reptiles that move slowly ("*lerdos*") are used to calm people ("*lerdar*"). For example, the products produced from *U. superciliosus *(dust and water) are used to "*amansar*" (to calm an aggressive person or ease the anger of someone betrayed by their wife or husband) [142]. There is an association between the biological characteristics of a lizard and the effects its use is expected to generate [134]. This observation is similar to that of Radbill [203] who pointed out that in homeopathic or imitative magic, it is assumed that certain qualities attributed to animals can be transferred to humans, and that this transfer can occur by inhalation, ingestion or application of the body parts of those animals.

It is worth mentioning that such "natural modeling" does not necessarily preclude empirical efficacy. In Oxchuc, México, the two most common medicines given to speed delivery in cases of protracted labor are made from the toasted tail and shell of the nine-banded armadillo (*Dasypus novemcinctus*) and the tail of the Virginia opossum (*Didelphis virginiana*) [40]. Both animals are characterized by odd reproductive habits: Nine-banded armadillos regularly give birth to litters of identical quadruplets, and the opossum produces 10 to 20 offspring per year (no doubt owing to its 13-day gestation period, one of the shortest in the animal kingdom [204]. While these reproductive anomalies undoubtedly influenced their therapeutic use, the tail of the Virginia opossum has demonstrated uterotonic action in recent laboratory and clinical trials. This action probably derives from the presence of prostaglandins, which are known to be oxytocic in very small doses [205].

Some diseases affect both animals and humans and can be treated with similar remedies. This way, some animals are used in ethnoveterinary medicine and are also used for the treatment of human diseases. Barboza et al. [193] and Souto et al. [62] recorded the utilization of animals (zootherapeutics) as sources of medicines in folk veterinary medicine in semiarid northeast Brazil and verified that 46 animal species (43 vertebrates and 3 invertebrates) are used in the prevention or treatment of veterinary diseases in that region. Souto et al. [62] pointed that parallels between zootherapeutic practices in human and animal ethnomedicine not only include the types of animals used and the prevalence of use of those wildlife resources, but also in the modes of administration of these remedies and the ethnomedical techniques employed. This view of ethnomedicinal symbioses was highlighted by McCorkle and Martin [206], who noted that nearly all the ways in which ethnopharmaceuticals are administered (both externally and internally) are shared in animal and human treatments. Souto et al [62] recorded that local residents in the semi-arid region of Brazil administer zootherapeutic medicines topically (powders, ointment of fats, and others), or orally to their livestock and pets in a manner very similar to human Complementary Alternative Medicine practices. The main techniques of preparing and administering animal-based remedies in local traditional medicine systems are commonly reported in Complementary and Alternative Medicinal practices in most parts of Brazil [7-9,31,132]. The close relationships between ethnoveterinary and human ethnomedicines can be easily explained from this perspective, as the main stock animals (e.g. cattle, sheep, goats, pigs, etc.) are mammals [132] that often have health problems very similar to humans and identical symptoms [62].

## Cultural beliefs

Historically, traditional cultures recognized the importance of belief and expectancy within the healing encounter and created complex rituals and ceremonies designed to elicit or foster the expectancy and participation of healer and patient, as well as the community as a whole. Spiritual healing techniques have been a fundamental component of the healing rituals of virtually all societies since the advent of man [207,208].

Folk-illnesses exist within the cultures that create them, "etiology, diagnosis, preventive measures and regimens of healing" [209] being provided by the culture. Cultural issues are recognized as important components of the provision of effective health care [210]. As noted by Straker [211], the supernatural world is as relevant in the diagnosis of illness as the natural world, with the main causes of illness being magical, mystical and animistic forces. Maher [212] showed that Aboriginal people have categorized illness as natural, environmental, direct supernatural, indirect supernatural and emergent or western causes. Pieroni and Quave [213] found, in a study carried at Ginestra/Zhure, Italy, that the aetiologies of various folk-illness are commonly linked to spiritual transmission, and that the treatments were often magical or psychotherapeutic in nature. Furthermore, for many illnesses deriving from relations with the supernatural, modern medicine is considered ineffective[214].

Besides their role in healing, natural products often have magical-religious significance, reflecting the different views of health and disease that exist within different cultures. In this context, animal parts are used to prepare clinical remedies as well as to make amulets or charms used in magical/religious diagnoses. Popular beliefs usually affect the way species are used in zootherapy [7,8,29,134,146]. One form of spiritual treatment involves the use of amulets containing reptile parts to protect the user from the "evil-eye" or from diseases. An example is caiman teeth (*C. latirostris*, *M. niger*, and *P. palpebrosus*) used as protection against snake bites [8].

The influence of magical beliefs integrated into traditional medicine is apparent [8]. In Brazil, for example, different animal species are used in magical-religious practices of Afro-Brazilian cults [29,65,142,215,216] in the context of rituals that emphasize the holistic nature of traditional medicine and that are designed to address spiritual, physical, and social-psychological problems [42]. Because medical systems are organized as cultural systems, the use of animal substances should be understood according to a cultural perspective [64].

## Socio-economic aspects of zootherapy

In addition to the belief systems, socioeconomic aspects also influence zootherapeutic practices [8]. Latin America has one of the greatest disparities in income distribution in the world. Overall, the health profile of the Latin American population can be classified as undergoing a slow epidemiological transition. At one extreme of the spectrum there is a high incidence of (and mortality from) chronic noninfectious diseases such as cardiovascular problems and cancer, which predominate in large metropolitan areas. On the other hand, infectious diseases still impose a heavy burden on the poverty-stricken parts of the population. The reasons for this dichotomy are two-fold: uneven socioeconomic development within countries and the extreme diversity of regional environments [217].

Studies suggest that Indigenous peoples of Latin America still have inadequate access to mainstream health services, and health prevention and promotion programmes, and that services that do exist are often culturally inappropriate [218,219]. The traditional medicine is widely available and affordable, yet in remote areas, and generally accessible to most people. In many developing countries, a large part of the population, especially in rural areas, depends mainly on traditional medicine for their primary health care, because it is cheaper and more accessible than orthodox medicine [8]. Traditional medicine is also more acceptable because it blends readily into the peoples' socio-cultural belief system [220-222]. Hence, resorting to the use of medicinal animals and plants, which were easily accessible and relatively cheap is an important component to healthcare in these gettings [8].

## Health Concerns

Traditional drugs and traditional medicine in general represent a still poorly explored field of research in terms of therapeutic potential or clinical evaluation. There is a current preoccupation about this, since it is well-established that all sorts of vegetable, animal and mineral remedies used in a traditional setting are capable of producing serious adverse reactions. It is essential, however, that traditional drug therapies be submitted to an appropriate benefit/risk analysis. De Smet [223] draws attention to the fact that some side-effects of traditional medicines have proven to be more severe than the disease that they are treating. Such considerations must be taken into account, further highlighting the need for clinical studies of traditional remedies.

Numerous infectious diseases can be transmitted from animals to humans [224]. Zoonoses constitute other animal health problems that affect public health. These represent an important threat for the welfare of human populations. In the urban and rural areas of the countries under development, zoonoses continue to present high frequencies [225]. In this context, the possibility of transmitting infections or ailments from animal preparations to the patient should be seriously considered [224]. Several organs and tissues including bones and bile can be a source of *Salmonella *infection causing chronic diarrhoea and endotoxic shock. The possibility of transmission of other serious and widespread zoonoses such as tuberculosis or rabies should be considered whenever animal tissues from unknown sources are handled and used as remedies [226].

Several species of animals have become a favorite target of hunters because its bones, organs and tissues are used in traditional medicine [136,143]. Indeed, numerous species of animals are hunted (poached) rather for their meat than for their supposed medical use [136,140]. Nevertheless, there is often an overlap between the two purposes, and transmission of disease can occur in both instances. One excellent example of this regards the primates [140]. Several species of monkeys have been identified as harboring infectious diseases transmissible to man with potentially grave consequences [224,227]. The related risks can be illustrated by a recent case from a Safari Park in Great Britain, where a colony of otherwise clinically healthy Macaque monkeys had to be humanely destroyed once it had been discovered that the monkeys harbored the simian herpes B virus [228]. While the agent is not harmful to monkeys, it causes mortality in 80% of affected humans. Certain researchers seriously consider the hypothesis that the worldwide epidemic of the HIV virus (causing the AIDS infection in humans) might have been initiated by transmission of a virus from monkeys several decades earlier [229].

The effectiveness of most of the medicines from wild animals/wild animal parts has not been scientifically studied and proven and their potency in many cases may be questionable. As pointed out by Pieroni *et al*. [230] the chemical constituents and pharmacological actions of some animal products are already known to some extent and ethnopharmacological studies focused on animal remedies could be very important in order to clarify the eventual therapeutic usefulness of this class of biological remedies. However, research with therapeutic purposes into the products of the animal kingdom has been neglected until recently [25,231]. In comparison to research done on plant drugs, modern pharmacology has done far fewer studies on animal products and minerals [232].

Historically, traditional use of plants as medicines has been extensively recognized, studied, and reviewed. However, such attention has not been paid to the potential of animals as a source of medicinally relevant substances. A excellent example of this potential is provided by insects. Trowell [233] points out that there are at least 16 times as many insect species as there are plant species, yet plant chemistry has been studied 7000 times as much as insect chemistry when comparing the amount of research per species. Nonetheless, the vast biodiversity which exists in the arthropod world, compared to all other organisms on earth, certainly suggests that arthropods should be given a more serious look [234]. Nevertheless, in last years, there has been increasing attention paid to animals, both vertebrates and invertebrates, as sources for new medicines [235].

## Environmental Approach

Various authors have discussed the conservation implications of the use of medicinal products derived from wild animal species [16,33,34,143,236]. In Latin America, as evidenced in the present review, the medicinal fauna is largely based on wild animals, including many endangered species.

The use of medicinal animals is common in both rural and urban areas. Biological remedies are openly commercialized in towns and cities, principally in public markets. It is common to find specific places in these markets where plants and animals are sold for medicinal purposes [31,32,38,42,66,67,131,133,135,138,144,237]. The commercialization of animals for medicinal purposes is a widespread phenomenon, with significant implications for their conservation and sustainable use [1]. Previous authors [238-242] have suggested that market expansion induces people to make greater use of wild animals for traditional medicine and that the practice has spread in developed nations of Asia and the Pacific (e.g., Taiwan, Australia). But other research suggests that the increasing use of animals for traditional medicine can also take place without economic prosperity. For example, Kritsky [242] reported that the use of insects for traditional medicine in China increased during the Cultural Revolution. Based on an ethno-zoological survey of the use of medicinal birds, Joseph [243] concludes that the use of birds to treat human ailments increased in Madhya Pradesh, Central India, because people could not afford modern treatments.

The worldwide market for animal parts and their medicinal derivatives is contributing to the loss of some species. The increased use of medicinal animals has led to over-exploitation of species like rhinos, tigers, musk deer, bears, monkeys and pangolins. In spite of international regulations and several national laws against poaching and heavy penalties for culprits, the extremely high prices offered for the parts of some species serve as strong incentives for illegal trade in animal parts to flourish [1,2].

It must be emphasized, however, that many factors affect animal populations in the world, and the use of these animals for medicinal purposes is only part of the problem. As such, the medicinal use of animals must be considered together with other anthropogenic pressures, such as habitat loss. The depletion of medicinal resources not only poses a challenge for conservation but represents a serious threat to the health of many human communities, and that efforts to stabilize the status of these species are important not only to conservationists but to millions of people whose health depends of the use of traditional remedies [136,140,224]. Moura and Marques [74] pointed that one characteristic in common among all zootherapeutic products, whether whole animals or their parts, is their lack of use for other purposes. In this sense, it is remarkable that in most cases, the medicinal products of animals are by-products from animals hunted for other purposes; thus, these multiple uses (including medicinal) of fauna and their impact on animal populations must be properly assessed and taken into consideration when implementing recovery plans for these species, especially those that are highly exploited [7-9,31,140]. Medicinal species whose conservation status is a cause of concern should receive urgent attention, and aspects such as habitat loss/alteration should be discussed in connection with present and future use of these species in folk medicine [8]

Zootherapy is intertwined with sociocultural and religious beliefs that must be understood by those engaged in modern conservation and protection of biodiversity. Celso [244] pointed out that natural medicine is one important use of biodiversity. Some traditional medicinal systems, like the Chinese Traditional Medicine, is recognized by the World Health Organization (WHO) and accepted by one-fourth of the world human population, and the reliance on traditional medicinal uses of animals by communities around the world should be addressed when designing strategies to conserve biodiversity. Conservation permits the continuing use of the resources in ways that are non-destructive and sustainable, while from the pharmaceutical point of view, it provides time to eventually demonstrate fully the potentially medicinal value of the resources [245].

Connections between traditional medicine, biodiversity and human health have recently been addressed by different authors [1,22,246-248]; have drawn attention to the fact that biodiversity loss can have indirect and direct effects on human well-being as well. The reliance on traditional uses of animals as food and as medicine by communities around the world highlights the need for further interdisciplinary research in ethnozoology which can be used in strategies to conserve biodiversity [53,249]. Furthermore, loss of wildlife resources, aside from threatening people's health and well-being, affects their cultural integrity. In Latin America, despite the many individual efforts of the governments to preserve the biodiversity for future generations, traditional knowledge, especially that derived from indigenous knowledge (such as Traditional Medicine), is also disappearing [45]. In this sense, understanding the contexts of traditional therapeutic uses of animals, is central for elucidating their potential impact in public health and biodiversity conservation.

## Conclusions

Latin America has a wealth of biological resources and is home to a large number of different ethnic and cultural groups, many of which have developed their own, distinct health care systems. As a result, the region is rich in traditional medicinal knowledge and zootherapy represents an alternative to official medicinal practices in rural areas and has also become part of urban popular medicine. Our results reveal that at least 584 animals are used for medicinal purposes in Latin America, underlining the importance of zootherapy as alternative therapeutic in region.

Animals provide the raw materials for remedies used to treat physical and/or spiritual diseases. Besides being influenced by cultural aspects, the relations between humans and biodiversity in the form of zootherapeutic practices are conditioned by the social and economic relations between humans themselves. In a region like Latin America, where the majority of the population has no access allopathic medicine, local medicinal animals and plant knowledge systems is of significance. The population uses traditional medicine due to the relatively low cost and difficult access to modern health facilities. Nevertheless, the interest in and intrinsic value of zootherapy not be only be attributed to the lack of access to modern medicinal services. Even in cities where modern health services are more accessible and specialized; many people continue to go to traditional healers showing the cultural acceptability of such practices.

Threatened species represented important medicinal resources in Latin America. This shows the need to integrate traditional knowledge into strategies to conserve and manage faunistic resources. Sustainability of harvesting of medicinal animals is challenged by many factors, from both social and ecological perspectives. It is important to respect differing views of the value of wildlife, while, at the same time, conserving biodiversity.

Using animal products as components of bioprospecting has implications for medicine, the environment, economy, public health and culture. Although widely diffused, zootherapeutic practices remain virtually unstudied, and so far there has been neither a demonstration of the clinical efficacy of the popularly used remedies nor an evaluation of the sanitary implications of the prescription of animal products for the treatment of diseases in the Latin America. New studies of medicinal fauna, which seek a better understanding of this form of therapy - including ecological, cultural and pharmacological aspects, are necessary.

## Competing interests

The authors declare that they have no competing interests.

## Authors' contributions

RRNA and HNA worked in the bibliographical classification, conception and the article final composition. The authors read and approved the final manuscript.
